# CUBIC: an atlas of genetic architecture promises directed maize improvement

**DOI:** 10.1186/s13059-020-1930-x

**Published:** 2020-01-24

**Authors:** Hai-Jun Liu, Xiaqing Wang, Yingjie Xiao, Jingyun Luo, Feng Qiao, Wenyu Yang, Ruyang Zhang, Yijiang Meng, Jiamin Sun, Shijuan Yan, Yong Peng, Luyao Niu, Liumei Jian, Wei Song, Jiali Yan, Chunhui Li, Yanxin Zhao, Ya Liu, Marilyn L. Warburton, Jiuran Zhao, Jianbing Yan

**Affiliations:** 1 National Key Laboratory of Crop Genetic Improvement, Huazhong Agricultural University, Wuhan, 430070 China; 2 Beijing Key Laboratory of Maize DNA Fingerprinting and Molecular Breeding, Beijing Academy of Agriculture & Forestry Sciences, Beijing, 100097 China; 3 Sanming Academy of Agricultural Sciences, Sanming, 365509 Fujian China; 4 College of Science, Huazhong Agricultural University, Wuhan, 430070 China; 5 College of Life Science, Hebei Agricultural University, Baoding, 071001 China; 6 Agro-biological Gene Research Center, Guangdong Academy of Agricultural Sciences, Tianhe District, Guangzhou, 510640 China; 7 Corn Host Plant Resistance Research Unit, United States Department of Agriculture-Agricultural Research Service, Box 9555, Mississippi State, MS 39762 USA

**Keywords:** Population development, Genome-wide association mapping, Cross-omics, Functional genomics, *Zea mays*

## Abstract

**Background:**

Identifying genotype-phenotype links and causative genes from quantitative trait loci (QTL) is challenging for complex agronomically important traits. To accelerate maize gene discovery and breeding, we present the Complete-diallel design plus Unbalanced Breeding-like Inter-Cross (CUBIC) population, consisting of 1404 individuals created by extensively inter-crossing 24 widely used Chinese maize founders.

**Results:**

Hundreds of QTL for 23 agronomic traits are uncovered with 14 million high-quality SNPs and a high-resolution identity-by-descent map, which account for an average of 75% of the heritability for each trait. We find epistasis contributes to phenotypic variance widely. Integrative cross-population analysis and cross-omics mapping allow effective and rapid discovery of underlying genes, validated here with a case study on leaf width.

**Conclusions:**

Through the integration of experimental genetics and genomics, our study provides useful resources and gene mining strategies to explore complex quantitative traits.

## Background

Plant breeding has had an enormous impact on food security and will continue to play a substantial role in the foreseeable future. Maize (*Zea mays*) is one of the most diverse crop species and a plant model in genetic studies; globally, it is also the most widely planted crop and an essential component in feeding an increasing world population. Researches have paid particular attention to maize functional gene discovery with an expectation to accelerate genetic improvement. Genome-wide association study (GWAS) has become a routine tool to study genotype-phenotype links [1, 2] and is especially suitable for maize due to abundant genetic diversity, rapid linkage disequilibrium (LD) decay, plentiful germplasm resources, and suitability for repeated phenotypic trials [3, 4]. While a very large sample size is necessary to obtain adequate power in GWAS for human genetics, the use of artificially designed populations with balanced allele frequencies and controlled population structure maintains statistical power with smaller populations in plant genetics. The Multi-parent Advanced Generation Inter-Cross (MAGIC) design is popular for identifying QTL of agriculturally important traits in crop plants [5–9] and has been considered as a next-generation permanent population [10]. For applied research, most breeders favor the diallel cross design, a classical mating scheme in which all parents in a set are crossed in all possible combinations to make hybrids, in order to explore the genetic underpinnings of traits, including general and specific combining ability [11, 12].

**Fig. 1:**
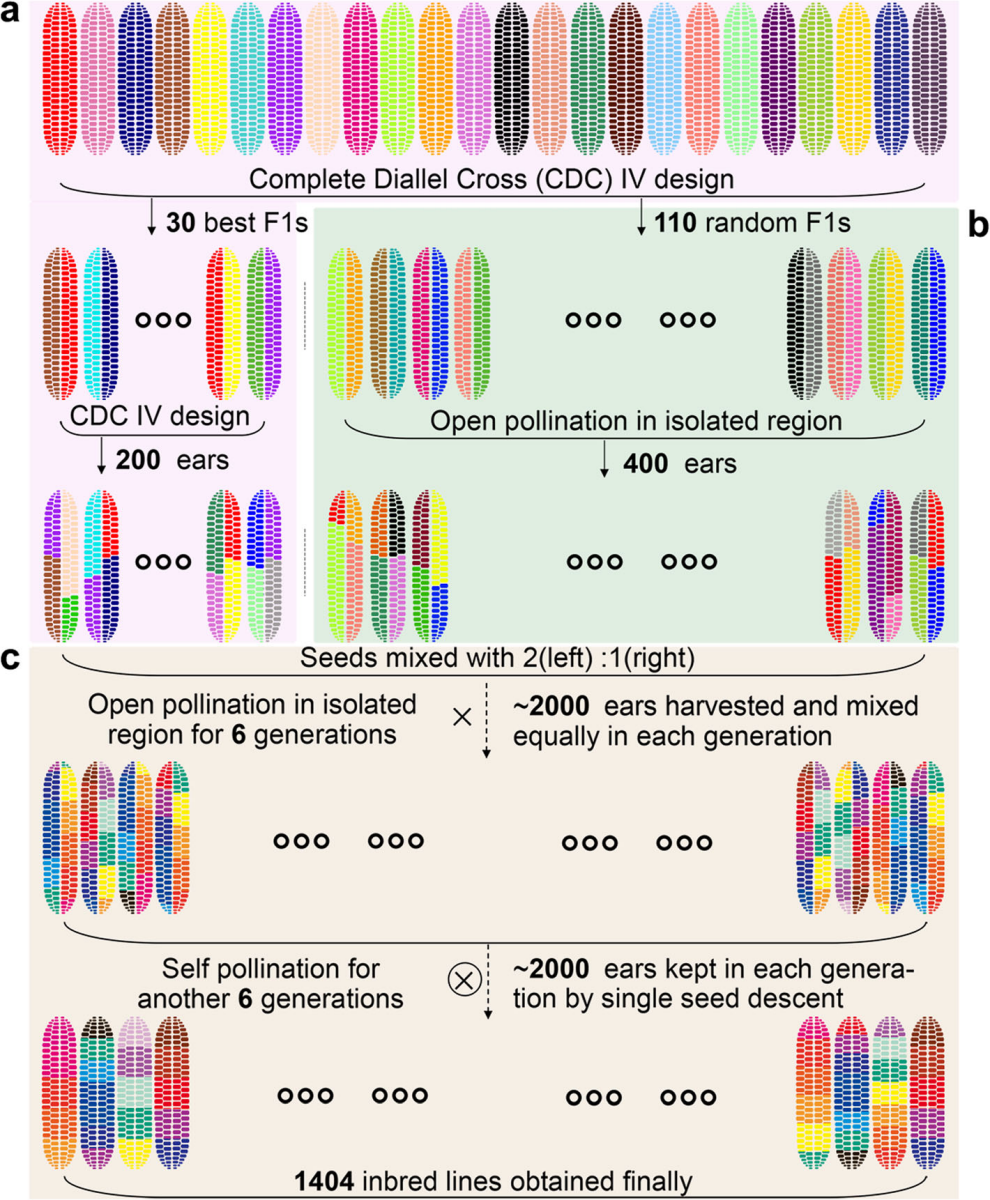
Development of the CUBIC population. The present CUBIC population, consisting of 1404 progenies, was derived from 24 elite Chinese maize inbred lines. **a** These 24 founders were crossed under a Complete Diallel Cross type IV (CDC IV) mating design, omitting parents and reciprocal crosses. Thirty F_1_s with best agronomic performance (early flowering time, small ear height, and big ear size) were selected to further cross in the CDC IV design. **b** Another 110 F_1_s were randomly selected for open pollination in isolation. Two hundred and 400 ears were harvested from the agronomically selected and randomly selected subsets, respectively, and seeds from the above F_1_s were mixed together in a 2:1 ratio with the expectation of improving population performance and maximizing diversity. **c** The resulting individuals were planted under open pollination in isolation for 6 generations. About 2000 ears of the most diverse lines were retained and mixed equally in each generation. Finally, the population was self-pollinated by single seed descent for another 6 generations, and a total of 1664 inbred lines were obtained, of which 1404 have been successfully sampled and sequenced, and thus used in further analysis

The identification of functional genes and favorable alleles is most useful for future breeding progress. With this in mind, and with the expectation to explore and exploit breeding resources, we describe the development of a Complete-diallel plus Unbalanced Breeding-derived Inter-Cross (CUBIC) population (Fig. 1). Here, we present the power of integrated GWAS and QTL mapping based on high-density marker coverage and extensive phenotyping of the CUBIC population and show how the uncovered favorable alleles can be modeled for directed inbred design. Many QTL regions were narrowed to a few candidates or to a single causal gene using information from multiple omics studies of the population. Epistasis was found to be prevalent and contributing to phenotypic variance, explaining up to 15% of trait heritability on average. The full exploration of genetic architecture of the 23 agronomic traits allowed genomics-directed maize improvement.

## Results

### Population design and genetic and phenotypic diversity

The CUBIC population consists of 1404 progenies descended from 24 elite inbred lines (Fig. 1). The 24 founders were selected from 4 subgroups and have been widely used in Chinese breeding over the past century (Additional file 1). The population was firstly derived from an existing breeding design, by applying 2-round diallel cross to 24 founder lines to select 30 F_1_ and 200 F_2_ hybrid lines with “favorable” performance (Fig. 1a), including early flowering, low ear height, large ear size, biotic and abiotic resistance, and other favorable agronomic traits. To make it suitable to comprehensively explore the genetic diversity among elite lines, genetic architecture of important agronomic traits, and favorable haplotypes from distinct elite founders, we borrowed and extended the population by integrating another 110 random F_1_s and open-pollinated 400 F_2_s (Fig. 1b). Seeds from the two sets of F_2_s were mixed with a ratio of 2:1, followed with 6 generations of open pollination and another 6 self-crossing generations by single seed descent (Fig. 1c). Additional to the breeding materials derived, CUBIC design generally descends from the traditional MAGIC design with the integration of an initial two round diallel crosses. This adjustment allows escape from arbitrary founder number and saves period of population development.

All progenies (including 3 progenies entered twice for replication) were re-sequenced with ~ 1× coverage and the 24 founders with 11× coverage; 194 lines were further genotyped using a maize200K array to cross-validate variant discovery. Following sequencing, variant calling, and imputation, approximately 5 Tb of raw sequencing data were collected (Additional file 2, Additional file 3: Figure S1) to characterize over 14 million high-quality SNPs and 439K short insertions and deletions (InDels). The 1404 lines and 30 checks (planted in every 50 plots), together with all founders, were evaluated in 5 locations (Additional file 3: Figure S2) for 23 agronomic traits (Additional file 4). The best linear unbiased predictor (BLUP) values for each line were used to reduce environmental noise in the phenotypic data. The CUBIC parents were highly diverse for the 23 traits of interest, as the variance of each trait was high and comparable to a natural population (Additional file 3: Table S1). In the entire CUBIC population, the 23 traits exhibited an average broad-sense heritability of 0.83, ranging from 0.72 to 0.93 (Additional file 3: Table S1), indicative of highly repeatable data, despite the polygenic nature of these traits.

### Single-variant-based association mapping explains limited heritability

**Fig. 2:**
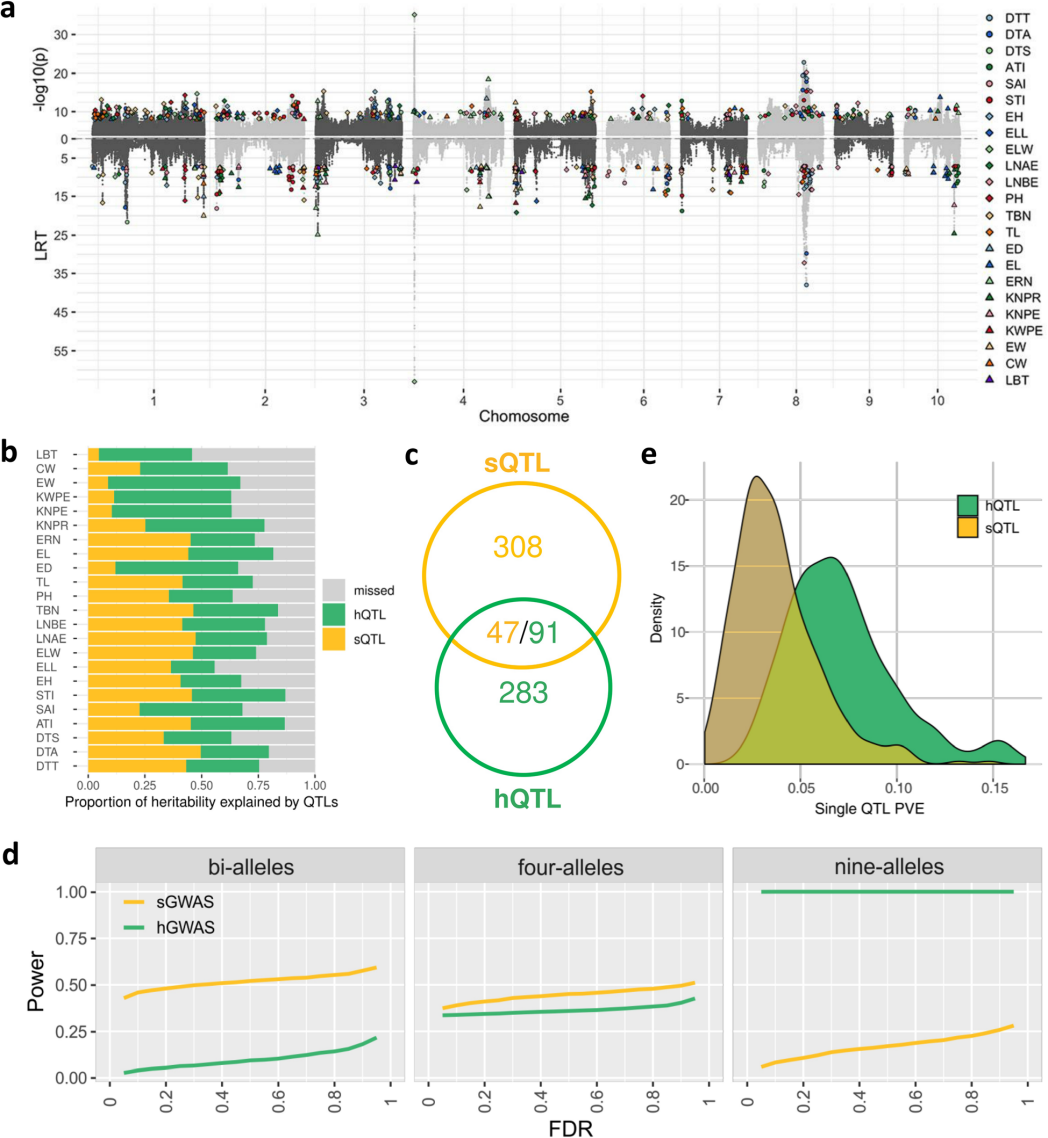
Overview of the 2 methods of GWAS analysis. **a** Summary porcupine plot of mapping results for 23 agronomic traits. Significant SNPs (*P* ≤ 1.23E−8) or bin (LRT value ≥ 7.1) at each QTL is marked by a dot, with each color corresponding to a trait. The abbreviations are defined in the “Methods” section. **b** The partition of heritability explained by all identified QTL. Each horizontal bar indicates heritability accounted for by sQTL (yellow), additionally by hQTL (green), and missing or unexplained (gray) relative to total heritability. **c** Venn diagram of co-localization between sQTL and hQTL, summed over traits. **d** Simulation analysis of mapping power under three QTL types. The three types of QTL were simulated to express as bi-allelic, four allelic, and nine allelic QTL, which were assumed to be produced by one, two, or 3 independent functional variants available in the local QTL region. The details of simulation analysis can be seen in the “Methods” section. **e** Comparison of variance explained (PVE) by single QTL identified by sGWAS vs. hGWAS

The CUBIC population showed weak population stratification and rapid LD decay (Additional file 3: Figure S3), which are promising for association analysis. Single-variant-based GWAS (sGWAS) using 11.8 million SNPs of minor allele frequency (MAF) greater than 0.02 was performed based on a mixed linear model (see the “Methods” section). This analysis detected 355 significant (*P* ≤ 1.23E−8) loci (hereafter called sQTL) for the 23 agronomic traits (Fig. 2a; Additional file 3: Figure S4; Additional file 5). Taking flowering time as an example, a total of 54 QTL were identified as associated with 3 flowering time traits (days to anthesis (DTA), days to tasseling (DTT), and days to silking (DTS)). Several previously cloned maize flowering genes (including *ZCN8*, *ZCN12*, *FLK*, *ZmMADS69*, and *VGT1*) and homologous loci validated in rice and *Arabidopsis* flowering (e.g., *HD1*, *VOZ1*, *HEX5*) were identified in these QTL intervals. Although the flowering time traits in maize, rice, and *Arabidopsis* are highly divergent [3], the genes identified in model plant species provide essential clues for exploring their roles in maize. The genetic architecture for male (DTA and DTT) flowering traits was similar but distinct from the female (DTS) flowering time trait (Additional file 3: Figure S4).

Despite the many QTL identified, most of them had a moderate additive effect, ranging from 0.01 to 0.83 standard deviations for each trait, with an average of 0.24. However, yield traits displayed weak effects overall, which was expected from the highly polygenic nature of such traits. Of the many QTL identified via sGWAS, most explained only a small proportion of the phenotypic variance, averaging 3.8%; however, seven contributed over 10% of the trait variance per sQTL. The sQTL jointly explained only 28% of the phenotypic variance on average (3.4~42.6%; Fig. 2b; Additional file 3: Table S2), far short of the estimated heritability for each trait.

### Uncovering QTL allele series via identity-by-descent mapping remedies missing heritability

The traceable feature of the present design makes it possible to infer every genomic segment of each offspring descended from any given parent (Additional file 3: Figure S5) using a hidden Markov model (HMM) [13] (see the “Methods” section). A total of 207,853 identity by descent (IBD) segments were found in all 1404 lines, ranging from 94 to 294, with an average of 148 segments per line (Additional file 3: Figure S6). This indicates that the recombination per line is nearly 7.6-fold higher than in bi-parental mapping populations [14, 15], 1.7-fold higher than in the public maize MAGIC population [9], and that the total number of recombination events in CUBIC is 1.4-fold higher than in the maize NAM population [16], which will allow higher mapping resolution. The size of the IBD segments per line varied, with a median of 4.2 Mb, and the distance between the closest markers on adjacent IBD segments approximately followed a bimodal distribution with the most at 100 bp and 80 kb, respectively (Additional file 3: Figure S6c). The segments that failed to be traced to any specific parent covered approximately 3.2% of the maize genome. In these regions, the identity-by-state between multiple parents was high compared to the flanking regions, presumably reflecting the co-ancestral origins.

With “bin” defined as a genomic span in which no recombination happened in any inbred line, a total of 27,005 unique bins were identified (Additional file 6). Bins varied tremendously in size across the genome, with a median of 100 kb (Additional file 3: Figure S6c). Treating the IBD states as haplotypes for each bin, we proposed another GWAS method (hGWAS; see the “Methods” section). This identified 421 significantly (likelihood rate test, LRT ≥ 7.1) associated loci (hQTL; Fig. 2a; Additional file 3: Figure S7; Additional file 7), with 6~26 hQTL per trait. Single hQTL contributed between 2% and 16.7% of the phenotypic variance for each trait, with an average of 7.2%, and 55 hQTL explained more than 10% of the phenotypic variance.

Interestingly, few of the sQTL and hQTL co-localized physically (Fig. 2c), but sQTL and hQTL jointly accounted for an average of 71% of the estimated heritability (46~87% per trait) (Fig. 2b; Additional file 3: Table S2), implying that the two GWAS methods work in a complementary manner. A hypothesis-based simulation (see the “Methods” section) suggested that while sGWAS reached the highest power with biallelic QTL irrespective of effect size, hGWAS power increased with increasing numbers of alleles and was more powerful for identifying QTL with minor effects (Fig. 2d; see the “Methods” section). These results agreed with the empirical observation that hQTL explained nearly twice the heritability as sQTL (Fig. 2e). The integration of these identified QTL provides a practical opportunity for the precise customization of elite parental lines. Moving beyond the potential application for an individual trait, we now demonstrate the value of the ensemble model in pyramid breeding by measuring the phenotypic effect of each QTL on multiple traits (Additional file 3: Figure S8 and Supplementary Notes). However, actually uncovering the causal genes underlying these QTL will undoubtedly accelerate precision pyramid breeding.

### Epistasis adds another layer to trait variance

Epistasis represents a non-linear interaction between two or more segregating loci and contributes to quantitative traits by biologically plausible mechanisms [17]. However, arguments are still present on its prevalence and significance to trait variance. While epistasis is important for trait variance [17, 18] and heterosis [19, 20], its prevalence in maize trait architecture is usually thought to be small [21, 22] or of large effect only at specific loci [23, 24].

**Fig. 3:**
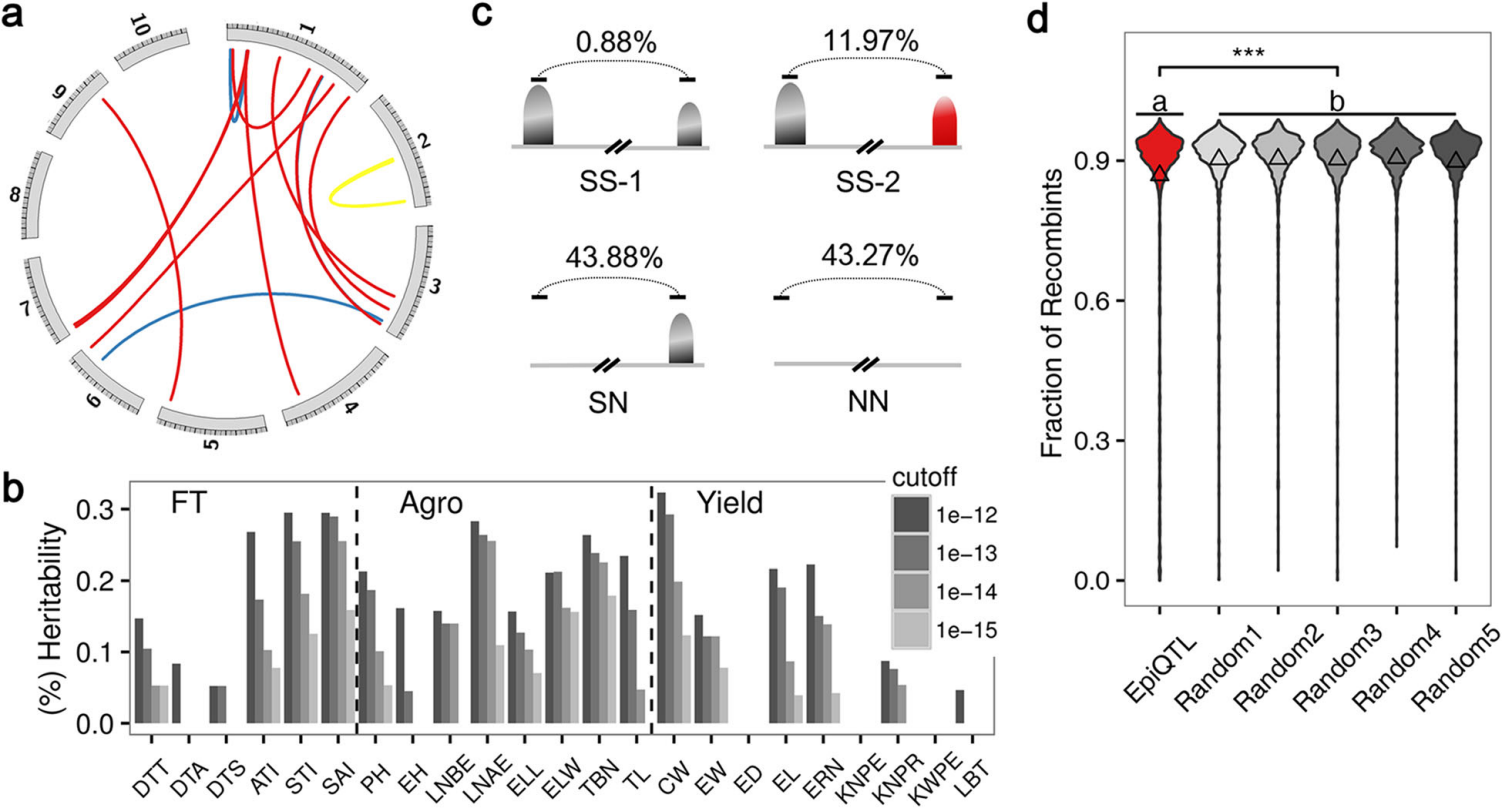
Identification of the epistatic contribution to trait variance. **a** Significant epistasis for flowering time traits. Other traits are shown in Additional file 3: Fig. S9. **b** Heritability explained by significant epistasis for different traits with different criteria averaged between 15.3% (*P* = 1E−12) and 4.8% (*P* = 1E−15). **c** Different epistatic combinations of loci and ratios for identified epiQTL: SS-1, two interacting loci linked with two sQTL affecting the same trait; SS-2, two interacting loci linked with two sQTL affecting different traits; SN, one of two interacting loci linked with a sQTL; NN, neither of two interacting loci linked with any measured sQTL. **d** Fraction of recombinants (combinations from different parents) encompassing interacting pairs of loci between epiQTL and random distal pairs

The balanced design and large population size of the present study provided a good opportunity to answer these questions. A total of 1466 significant epistatic interactions (epiQTL) were identified for 21 of the 23 measured traits and explained an average of 15.3% of the trait heritability, although this varies greatly between traits (Fig. 3a, b; Additional file 3: Figure S9; Additional file 8; see the “Methods” section). Most of the epiQTL were found with 1 of the 2 interacting loci linked with sQTL (43.88%) or neither of the 2 interacting loci linked with any sQTL (43.27%) (Fig. 3c). A subset (4.22%) of epiQTL was found co-occurring with previously identified functional networks (Additional file 3: Figure SS10), including protein-protein interactions (PPI) [25], co-expression relationships of protein and transcript levels [26], and the interactive genome architecture from ChiA-PET experiments on H3K4me3 modifications [27]. These multiple lines of evidence provide clues to understanding the molecular mechanism of epistasis. Interestingly, the recombination frequency in the epiQTL regions was significantly lower than random levels (Fig. 3d), suggesting that epiQTL regions were likely functional and experiencing selection.

### Use of founder genome assignments and multiple omics data for rapid gene mining—ear leaf width example

Among all detected QTL in the present study, about 10.4% harbored genes that had been cloned and 11% contained annotated candidate genes of phenotypic relevance, another 10% overlapped with QTL identified in previous studies, and the remaining 68.6% were newly identified (Additional files 5 and 7). Additionally, 57 QTL were located in non-genic regions, of which 89.7% were identified close to MNase hyposensitive (HS) proximal regions [28], and 53.4% and 55.1% resided in accessible chromatin regions identified in leaf and inflorescence [29], all were significantly higher than expected by chance (Additional file 3: Figure S11). This strongly suggests the importance of non-coding sequences that may influence phenotypic variation by regulating other genes.

**Fig. 4:**
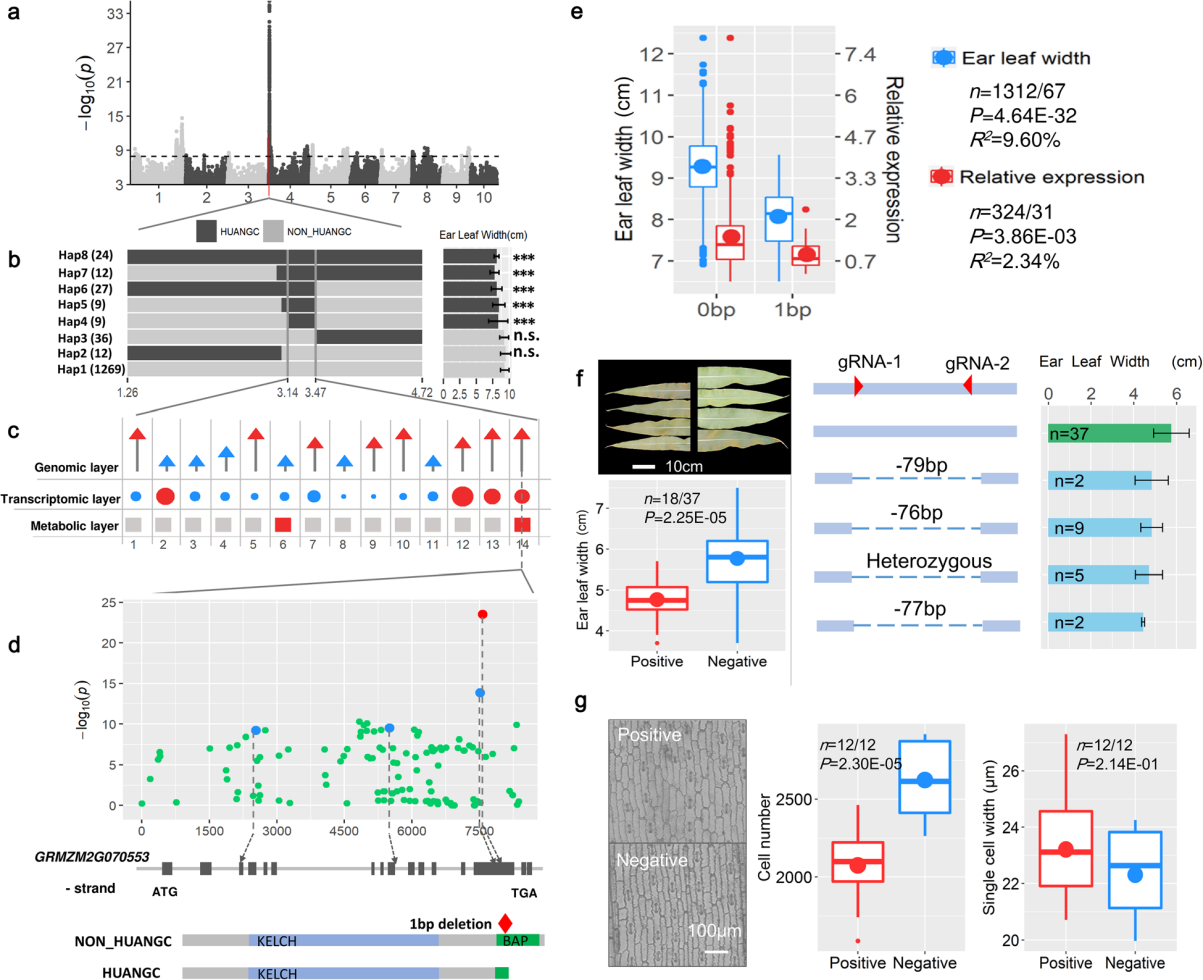
Integrating omics data empowers rapid gene mining for ear leaf width. **a** Manhattan plot of ear leaf width (ELW) based on sGWAS. The black dashed line represents the cutoff of 1.23E−8 based on adjusted Bonferroni correction. **b** Refinement of the major ELW QTL on the short arm of chromosome 4. The left panel illustrates the 7 major haplotypes (*n* > 8) at this QTL within the original 3.5-Mb interval. This test enabled the QTL to be delimited to a 334-Kb region with 14 genes. **c** Determination of candidate gene via omics data. The upper, middle, and bottom panels are the genomic, transcriptomic, and metabolic levels, respectively. The 14 genes are sequentially ordered based on physical positions (Additional file 9), and the genes associated with red symbols imply the candidate genes that influence the ear leaf at the different levels. **d** Local Manhattan plot within *ZmGalOx1*. Four type I polymorphisms were identified at this gene. The type I InDel is colored in red and SNPs are colored in blue; the bottom panel is a protein structure corresponding to the 1-bp InDel mutation. **e** Genetic impact of the 1-bp InDel on ELW and *ZmGalOx1* expression. The *P* and *R*^2^ values were calculated using ANOVA. **f** Functional validation of *ZmGalOx1* via CRISPR/Cas9. **g** Cytological experiment of CRISPR/Cas9 modified *ZmGalOx1*-carrying lines. The epidermal cells in the abaxial leaf surface for the CRISPR/Cas9 edited lines were observed. The *P* values are based on *t* test, and the error bars in bar plots represents the standard deviation in **f** and **g**

Rapid identification of genes underlying novel QTL is challenging, and one targeted strategy in the present design uses linkage-like analysis to consider phenotypic differences between individuals with distinct allelic states. This is possible since the offspring panel approximates multiple RIL populations once founder contributions have been assigned to every progeny (see the “Methods” section) and allowed the QTL to be narrowed to very small regions. We present the ear leaf width (ELW) trait as an example, since one large effect QTL was identified on chromosome 4 by both GWAS methods (Fig. 4a). This QTL contributed to ~ 13% of the ELW variance, causing a decrease in ear leaf width of 0.7 cm, and the interval was estimated to between 1.26 and 4.72 Mb using local LD architecture, an interval containing 127 genes. By assessing the IBD effect spectrum on the basis of peak bin (see the “Methods” section), a total of 15 IBD states were observed in this region, and only 1 from HUANGC showed a statistical difference from the 14 other parental alleles for phenotypic effect (Additional file 3: Figure S12). The progeny individuals were thus grouped into 2 distinguishable alleles (HUANGC and NON-HUANGC), and comparing phenotypic difference against allele type narrowed the QTL to a 334-Kb region (*t* test, *P* < 0.01, Fig. 4b) in which there are only 14 genes (Additional file 9). Of these, 8 were highlighted due to the inclusion of loss-of-function (type I) and regulatory (type II) variants in the dataset (Fig. 4c; see the “Methods” section).

RNA-seq of 391 randomly selected offspring lines (using RNA from leaf tissue collected at the V9 stage) revealed that the expression of 4 genes, of all 14 candidates, was significantly correlated with ear leaf width variation (*P* < 0.01, Fig. 4c). A metabolic profile of these lines detected 13 metabolites correlated with trait variation (*P* < 0.05, Additional file 3: Table S3) from a total of 237 primary metabolites and 10 hormones identified in leaf tissue at the same period. Hypothesizing that the metabolites would mediate the link between the causal gene and phenotypic variation allowed us to determine that 2 of the 14 genes annotated closely to the differently expressed metabolites, particularly for sucrose metabolism (Fig. 4c; Additional file 9).

Considering all evidence together, the gene GRMZM2G070553 encoding galactose oxidase (named *ZmGalOx1*) was identified as a strong candidate for this QTL. *ZmGalOx1* contains 4 type-I polymorphisms, 3 SNPs, and a 1-bp InDel (InDel_1/0), which were confirmed by re-sequencing (Additional file 3: Table S4). The 1-bp InDel displayed the most significant association (*P* = 2.93E−24; Fig. 4d) and was highly significantly correlated with the trait differences and slightly related to the expression variation (Fig. 4e). Importantly, the deletion was present exclusively in the HUANGC allele (Additional file 3: Table S5) and will induce a truncated protein with an incomplete BAP domain, which has been reported to influence leaf development in tobacco [30]. *ZmGalOx1* was then knocked out by CRISPR/Cas9 in maize variety ZZC01, which displays wide leaves. A total of 55 transgenic T0 seedlings were obtained, of which 18 were confirmed by re-sequencing to have been edited in the target gene (positive control) and 37 seedlings had no sequence changes and were used as negative controls. The positive controls contained 4 editing types with various deletion lengths located upstream from InDel_1/0, and all expressed narrower ear leaves compared to the negative control lines (*P* = 2.22E−05, Fig. 4f). This confirmed the causal effect of *ZmGalOx1* on the ear leaf width. The positive control lines had significantly fewer cells (*P* = 2.30E−05), but no difference in single-cell width compared to non-edited ones (*P* = 0.14; Fig. 4g), which is consistent with the cytological results found in 59 lines from the CUBIC population with contrasting alleles of the 1-bp deletion (Additional file 3: Figure S13). Taken together, this data strongly implies that *ZmGalOx1* affects leaf development by regulating cell proliferation efficiency and that this gene is hypothetically involved in the glycolysis metabolic network (Additional file 3: FigureS14).

### Narrowing QTL regions to candidate causal genes via cross-omics mapping and cross-population analysis

Beyond the specific strategies that can be applied in the present population, we attempt to systemically identify functional genes within novel QTL by two general strategies. The first involves integrating transcriptomic data to perform omics QTL mapping, and the second uses sequencing of an independent population to provide a cross-population analysis (Additional file 3: Figure S15a-c).

Transcriptomic analysis has become a common tool for functional gene identification and to further interpret regulatory mechanisms associated with complex traits [4, 31, 32]. Here, cross-omics mapping (Additional file 3: Figure S15ab) was performed using the expression data from 391 progenies by integrating associations including expression-genotype (eQTL) and expression-phenotype (epQTL) links [32], together with the genotype-phenotype associations (pQTL) detailed above. All these association analyses were performed with mixed linear models (see the “Methods” section). As a result, eQTL from 674 genes showed co-positioning with 170 (47.9%) pQTL identified by sGWAS, reducing candidate number on average from the original 72.7 genes to 4.0 genes per QTL (Additional file 10). This result suggested key driver genes of the hypothesized genotype-expression-phenotype mechanism, including *RAP2* (AP2/EREBP transcription factor, GRMZM2G700665, Additional file 3: Figure S13de), *ZCN12* (GRMZM2G103666), and *zmBBX19* (B-box zinc finger protein 19, GRMZM2G422644) for male and female flowering interval, and the gibberellin receptor *GID1L2* (AC218900.3_FG001) for ear height (Additional file 3: Figure S15d; Additional file 3: Figure S16). Interestingly, a larger set of 280 (81.1%) pQTL from sGWAS displayed trans-eQTL effects regulating the expression of those genes outside pQTL intervals significantly (Additional file 11). This analysis produced more functional candidates that were not observed by phenotype-based GWAS. The epQTL adds indirect evidence of gene-expression-phenotype links (Additional file 12) and exactly verified some of the co-localization of pQTL and eQTL findings. The ratio will undoubtedly be increased when more tissues are used for the expression quantification. Interestingly, most (96.5%) epQTL were novel and did not cover any genotype-phenotype associations, suggesting direct expression-phenotype mapping is mutually complementary to conventional GWAS. These new associations included many known links, or some inferred from other species, such as *MADS4* (GRMZM2G032339), *ZMM15* (GRMZM2G553379), *VOZ5* (GRMZM2G449165), *NFC103B* (GRMZM2G320606), *HB120* (GRMZM2G056600), and *BHL32* (GRMZM2G180406) for flowering time traits (Additional file 3: Figure S17).

Furthermore, comparisons of GWAS observations across diverse populations have been shown to be valuable to both cross-validation and fine-mapping [33]. A collection of over 500 unrelated inbred lines has been widely used for QTL discovery [4] which was re-sequenced (~ 20× coverage) in the present study to integrate its complementary advantage of higher resolution mapping power compared with the CUBIC design. This was done by regarding all CUBIC QTL as candidate regions to perform association analysis with a liberal threshold (*P* < 1E−4) in the previous population. In total, 77 (10.6%) CUBIC QTL were co-mapped in the unrelated population (Additional file 13), allowing these candidate intervals to be narrowed. This analysis prioritized the association of *RAP2* (Additional file 3: Figure S15e) for flowering time, *PHD17* (E3 SUMO-protein ligase SIZ1, GRMZM2G155123) for ear height, and *ACCO35* (1-aminocyclopropane-1-carboxylate oxidase35, GRMZM2G052422) for tassel branch number. Together, combining these systematic analyses provided a general framework and an excellent resource for inference of functional candidates and insights into the mechanisms governing complex traits.

## Discussions

Identification of the genes controlling phenotypic variation aids in understanding the genetic basis of key traits and relevant crop improvement. Plant populations resulting from controlled crossing experiments provide an opportunity to facilitate statistical designs for GWAS and can improve mapping power [4]. In the present study, an innovative synthetic population (CUBIC) integrating advantages of both the diallel cross and MAGIC designs proved valuable in complex trait dissection thanks to its high phenotypic diversity, large population size, rapid LD decay, weak population structure, and traceable recombination events. The process of artificial selection was introduced in the early development of CUBIC population, which can enrich the favorable genes with low frequency, thus enhancing the QTL detection power and help to understand the breeding process in genome level. The contribution for each parent in genome level ranged between 0.5% for YUANFH and 13.4% for HUANGC (Additional file 3: Figure S18). As expected, the enriched genome segments for specific parent are not random and some of them overlapped with the mapped QTL (data not shown). The weighting toward material with desirable phenotypes of CUBIC helps make the resulting material more immediately relevant for breeders and the inclusion of more diverse germplasm ensures its effectiveness as a trait mapping resource. The IBD-based GWAS is complementary to conventional single-variant-based association mapping, and the former is particularly superior in the identification of QTL with allelic series. Almost 71% of the trait heritability was explained by hundreds of QTL uncovered by both mapping methods.

Beyond the great resources of genotype-phenotype associations, we further demonstrate how this population design is additionally valuable to narrow down the candidate regions due to its IBD traceable feature, and how integrative omics data to rapidly pinpoint causal genes responsible for phenotypes of interest, using ear leaf width as an example. Genomic, transcriptomic, and metabolic data were integrated to clone the causal gene, *ZmGalOx1*, responsible for ear leaf width, which has been verified with CRISPR/Cas9 experiment. This again reinforced the capacity of the CUBIC population as a tool to rapidly mine genes. Beyond this specific case, we took a further step to systematically identify likely functional candidates by cross-mapping and cross-omics analysis, creating useful resources to explore and understand physiological mechanisms of important agronomic traits.

## Conclusions

Future breeding can be transfigured to be more precise under the guidance of genomic information, by assembling major mapped QTL for a target trait into one breeding population and by pyramiding favorable QTL for multiple traits to optimize the overall performance using exact assessment of co-variation of diverse traits. We expect these substantial gene-trait links to provide guidance and resources for precise maize genetic improvement. With the creation of superior alleles at directive loci by emerging genome editing techniques (as pioneer explorations shown in [34–37]), we now move toward a knowledge-driven advanced crop improvement era.

## Methods

### Population design

The CUBIC design is a modified Multi-parent Advanced Generation Inter-Cross (MAGIC) population consisting of 1404 progeny. It was derived from 24 elite Chinese maize inbred lines from 4 divergent heterotic groups (Additional file 1), which included 4 founders from LvDaHongGu germplasm (旅大红骨种质): LV28 (旅28), E28, DAN340 (丹340), and F349; 4 founders from ZI330 germplasm (自330亚群种质): ZI330 (自330), ZONG3 (综3), ZONG31 (综31), and HUANGC (黄C); 15 founders from SiPingTou germplasm (四平头种质): HZS (黄早四), HYS (黄野四), TY4 (天涯4), YUANGFH (原辐黄), CHANG7-2 (昌7-2), K12, XI502 (西502), LX9801, H21, SHUANG741 (双741), Q1261, JI853 (吉853), JI53 (冀53), 5237, and 81515; and 1 founder from Yugoslavia-improved germplasm (南斯拉夫群体选系): NX110 (农系110). These 24 founders were crossed under a Complete Diallel Cross type IV (CDC IV) mating design, omitting parents and reciprocal crosses, in the summer of 2004. Thirty F_1_s with acceptable agronomic performance (early flowering, short ear height, and big ear size) were selected to cross further in the CDC IV design, and an additional 110 F_1_s were randomly selected to open pollinate in isolation in the winter of 2004. A total of 200 and 400 ears resulting from the best and the random crosses, respectively, were harvested. Seeds were mixed together in a 2:1 ratio (biased to improve population performance), and individuals were planted under open pollination from the summer of 2006 to the winter of 2008 in isolated regions for 6 generations. About 2000 ears of the most phenotypically diverse individuals were retained, and seeds were mixed in equal proportions in each generation. The population was then self-pollinated by single seed descent beginning in the summer of 2009 for another 6 generations. During the open- and self-pollinated processes, the greatest possible variation for several traits was constantly maintained to maximize the phenotypic diversity. A total of 1664 inbred lines were obtained, of which 1404 were successfully sampled and sequenced, and thus used in further analysis.

### Phenotyping

All 1404 inbreds were planted in the year of 2014 at 5 locations (Additional file 3: Figure S2; N 43° 42′, E 125° 18′, Yushu City, Jilin Province; N 42° 03′, E 123° 33′, Shenyang City, Liaoning Province; N 40° 10′, E 116° 21′, Changping District, Beijing City; N 38° 39′, E 115° 51′, Baoding City, Hebei Province; N 35° 27′, E 114° 01′, Xinxiang City, Henan Province) in Northern China, where the 24 elite founders that served as the parents of the population are the most adapted. The lines were planted with a random order at each environment to eliminate the potential confounding effect. About 17 individual plants were planted for each line, and the line Chang7-2 was planted after every 50th entry, whose phenotypes are used to correct for trend and spatial heterogeneity. Twenty-three phenotypes were measured at each location, including *6 flowering time traits*: days to tasseling (DTT, measured as the interval from sowing to the day the tassel appeared in half of the individuals per line), days to anthesis (DTA, measured as the interval from sowing to the day of pollen shed for half of the individuals), days to silking (DTS, measured as the interval from sowing to the day silks emerged for half the individuals), and the intervals between them: interval between anthesis and tasseling (ATI), interval between silking and tasseling (STI), interval between silking and anthesis (SAI); *8 developmental agronomic traits*: plant height (PH, vertical height from the ground to the top of the tassel, with an accuracy of 0.5 cm), ear height (EH, vertical height from the ground to the node where the top ear arises), ear leaf length (ELL, straight length of the first ear leaf), ear leaf width (ELW, width of the ear leaf measured at the widest point), leaf number above ear (LNAE, leaf or node number from ear leaf to the top; counted including the ear leaf), leaf number below ear (LNBE, leaf or node number below the ear leaf; not including the ear leaf), tassel branch number (TBN, only the primary tassels were considered), tassel length (TL, straight length of the main branch); *and 9 ear traits*: ear weight (EW), ear diameter (ED), ear length (EL), ear row number (ERN), kernel number per row (KNPR), kernel number per ear (KNPE), kernel weight per ear (KWPE), cob weight (CW), and length of the barren tip (LBT). For flowering times, all individuals in a line were considered, while only 5 consecutive plants in the middle of each line were measured and the average value was used for developmental agronomic traits. For measuring ear traits, 5 approximately equidimensional ears were randomly selected to represent each line.

For each trait, the variance of genotype and environment on phenotype were estimated, and the line mean-based broad-sense heritability for each trait was calculated as: ${H}^2={\delta}_g^2/\left({\delta}_g^2+{\delta}_e^2/n\right)$, where ${\delta}_g^2$ is the genetic variance, ${\delta}_e^2$ is the residual variance, and *n* is the number of environments. The estimates of ${\delta}_g^2$ and ${\delta}_e^2$ were obtained by the mixed linear model, treating genotype and environment as random effects. The best linear unbiased predictor (BLUP) value for each inbred line was calculated across all environments using the mixed linear model in the R package “lme4” [38]. The BLUP values were used in subsequent analyses, including basic phenotypic statistics, correlation analysis, and GWAS. The phenotypic variance or heritability explained by either sQTL or hQTL for a given trait was estimated in an analogous way by using linear regression, and the total variances explained by integrated methods were estimated jointly.

### Whole-genome sequencing (WGS)

#### Sample collection and DNA extraction

Young leaves in the vegetative growth stage were collected about 5 weeks after planting and flash-frozen in liquid nitrogen. Genomic DNA from each sample was extracted with the cetyltrimethylammonium bromide (CTAB) method [39] and dissolved in double-distilled water. The DNA was checked for quality and quantity on agarose gels and a Qubit fluorometer (Invitrogen). Samples having at least 1 μg of total DNA, A260/A230 above 2.0, and A260/A280 above 1.8 were selected to construct the sequencing library.

#### Library construction

High-quality genomic DNA was column-purified to remove protein, carbohydrates, salt, and other impurities. Purified genomic DNA (1 μg) was fragmented using a Biorupter UCD-200 (Diagenode). The fragmented DNA was inspected for quality via agarose gel electrophoresis, and fragments in the range of 300–500 bp were concentrated. DNA was repaired by adding adenine nucleotides (A) and a sequencing adapter to remove gaps at the 3′ end of the fragmented DNA; repaired DNA was then further purified by 2% agarose gel electrophoresis. DNA fragments of around 500 bp were recovered by PCR amplification and purification to obtain the final library, which was inspected again for quality by agarose gel electrophoresis and qPCR with the StepOne Plus Real-Time PCR system (ABI).

#### Sequencing

DNA libraries were sequenced with the Illumina HiSeq 2500 platform using V4 reagents with 125-bp paired-end reads, generating a total of 5 Tb of sequenced base pairs. The library preparation and sequencing work described above was accomplished by the BerryGenomics Company (Beijing, China).

### Variant discovery

#### Quality control of raw reads

Adaptor sequences were removed as the first step of the analysis, and low-quality reads were trimmed by Trimmomatic (Version 0.33) [40], using the following parameters: TRAILING = 3, MINLEN = 50, and HEADCROP = 10.

#### Read alignment

Clean reads were mapped to the maize B73 reference genome (v3.25, downloaded from http://plants.ensembl.org) using the BWA aligner (Version 0.7.12) [41], which displays a good balance between running time, memory usage, and accuracy. The FM-index for the reference genome was produced first, and reads for each sample were aligned against the reference using the “aln/sampe” option with default parameters. The unique mapped reads extracted from SAM files were sorted and indexed using Picard (Version 1.119) [42].

#### Insertion/deletion realignment and base quality recalibration

The RealignerTargetCreator and IndelRealigner modules from the Genome Analysis Toolkit (GATK, Version 3.5) [43] were used to perform local realignment around InDels to correct mapping artifacts. SAMtools (version 0.1.19) [44] and UnifiedGenotyper from GATK were first used to generate a high-quality SNP set as known sites to build the covariance model and estimate empirical base qualities for each individual. Next, BaseRecalibrator and PrintReads tools from GATK were used to recalibrate base quality scores in order to correct sequencing errors and other potential experimental artifacts.

#### Variant calling (SNPs and short insertions and deletions, InDels)

The recalibrated BAM files were processed with SAMtools and UnifiedGenotyper from GATK. For GATK, the parameter “-glm” was set as “BOTH” to obtain SNPs and InDels simultaneously. Variant calls from the SAMtools mpileup package were identified using default parameters. Variants identified by both programs were only kept if they satisfied mapping quality (MQ ≥ 20.0) and sequencing coverage (DP ≥ 2 and DP ≤ 100). The filtered variants of each sample were further combined by GATK CombineVariants, and another re-calling process of every individual using GATK UnifiedGenotyper was performed to obtain initially integrated genotypes at the population level. Next, variants with a missing rate greater than 98% were removed, and heterozygous sites with a “Lowqual” label were excluded. The resulting variant calls were stored in a variant call format (VCF).

#### Variant calling pipeline evaluation

To estimate the error rate of the variant calling pipeline for the present study (i.e,. for the maize genome), a number (10×) of pair-end sequencing reads were simulated from the reference genome with 0 miss-match and the same pipeline was utilized to call variants. Theoretically, no differences should be identified; such variants could only be derived from incorrect mapping and calling, which may occur due to intrinsic duplication of genome fragments. Only 12 heterozygous SNPs and 0 InDels were called using the simulated sequences. This indicated that our maize variant calling process applied above is reliable, and systematic errors (caused by the complex genome structure) are limited and most likely to happen for heterozygous loci. With the expectation that this kind of error would occur most frequently at specific sites, we further simulated the same procedure 10 times with the aim of covering the majority of this type of error. The variant sites identified from this analysis were removed, if present, from the variant set called from real data.

#### Genotyping by 200K Array

A subset of 194 lines, including 24 founders and 170 randomly selected progenies, were further genotyped using the Affymetrix Axiom Maize Genotyping Array 200K (from Peking University, China). The Affymetrix Power Tools (APT) and SNPolisher package [45] were used to analyze the genotyping data in the “cel” format. Briefly, the DQC value and call rate for each sample were set larger than or equal to 0.82 and 0.97, respectively, and acceptable poly high resolution (PHR) values were retained leading to a dataset of 53,831 high confidence SNPs. The average concordance rate between the 200K Array and the whole-genome sequencing was 97%. All SNPs obtained from re-sequencing and the 200K array were integrated to perform genotype imputation for all lines, and those inconsistent genotypes were considered as unknown.

### Genotype imputation

Missing genotypes were imputed using Beagle (version 4.0) [46]. Exploration of imputation accuracy with various missing data rates was done via the comparison between imputed calls and randomly masked genotypes (~ 5000,000 for site × individuals) for chromosome 10 only. The best parameter combination for the present study was determined to be as follows: window = 50,000, overlap = 5000, original missing rate < 75% (sites from the 200K array were always kept), and using the imputation-then-removing strategy of [47]. With this method, the average imputation accuracy was 98%. Ultimately, a set of 14,126,424 high-confidence SNPs and 439,670 InDels were retained for further analysis, covering about 99.13% of predicted maize genes (38,805 genes).

### Mosaic map tracing IBD origins from 24 founders for each progeny line

The hidden Markov model (HMM) raised in [13] was used to make a multipoint probabilistic reconstruction of the genome of each progeny line as a mosaic of the founder identity-by-state (IBD). Each progeny genome is made up of IBD segments of the founder genomes, with a transition between founders occurring whenever a recombination has occurred (Additional file 3: Figure S5). The biallelic SNPs cannot distinguish between all founders, so the HMM used the information of neighboring SNPs to compute the posterior probability, ${P}_{s,f}^i$, that at a given SNP locus *s*, the CUBIC progeny line *i* is descended from founder *f*. The IBD state is built as the founder with the maximum posterior probability ${\max}_f\left\{{P}_{s,f}^i\right\}$ at SNP locus *s* for progeny *i*, only if the maximum posterior exceeds twofold of expected value by chance (1/24); otherwise, it was considered an unknown state. Segments that failed to be traced to any specific founder covered approximately 3.1% of the maize genome. In these regions, the identity-by-state between multiple founders was highly comparable compared to the flanking regions, presumably reflecting the co-ancestral origins.

Upon the CUBIC design, the HMM made the following approximations and assumptions: (i) the genome of each progeny line is homozygous due to advanced selfing generations, so we filtered out the SNPs of high heterozygous genotypes (> 10%) or missing calling before imputation (> 25%), leaving ~ 240K high-quality SNPs for IBD reconstruction; (ii) the effective number of generations (*G*) approximately be 9, since the eight rounds of inter-cross generation (dialle cross or open pollination), plus one round of approximate cross-generation from six generations of selfing, because on average, only half recombination events are accumulated per selfing [48]; (iii) the identity of the founder in a given IBD segment in the mosaic is uncorrelated with other segments for that individual, and the length of segment follows an exponential distribution with a mean length *ρ*/*G*, where *ρ* is the genetic length of the segment, corresponding to a consensus map (Additional file 14) built with ten bi-parental linkage maps [15].

The power of the HMM method in correctly tracing the IBD origins of CUBIC progeny lines was evaluated by simulating pseudoprogeny lines by reshuffling 24 founder genomes. First, we set the number of recombinant segments of the progeny line as 180 (the median of recombination per line in real data) and to be proportional to the chromosome length. Second, we set the location of the break point based on the empirical recombination *ρ* from the consensus genetic map (Additional file 14). The 24 founders were then randomly assigned to the simulated segments for each line. The procedure repeated 100 times to simulate 100 pseudoprogeny lines. The simulated recombination count of the 100 pseudoprogeny lines appeared to apparently decreased recombination in centromere relative to the arms of chromosomes (Additional file 3: Figure S19a). The SNP genotypes of 100 pseudoprogeny lines were projected from the founder SNP genotypes in every simulated IBD segment. Then, for each pseudoline, the HMM with the same criteria in real data was run to trace back to the 24 founders. The power was defined as the proportion of SNPs across the genome that have the identical IBD origin to what it was simulated to be. It was found that the current procedure of the HMM approach had the power of 93–97% (along different chromosomes) to correctly identify the founder origins (Additional file 3: Figure S19b).

### Single-variant-based GWAS

In total, 11.8 million high-quality SNPs (MAF ≥ 0.02) were used to perform sGWAS. The relatedness matrix (*K*, random effect) was calculated using the “pylmmKinship.py” script from pylmm [49], and the top ten PCs (which explained 8.76% of the variance) were generated by GCTA [50] and applied as a fixed effect. The mixed linear model (MLM) corrected by a relatedness matrix and the top ten PCs was implemented by the software Tassel 3.0 [51]. The significance threshold for the association was set to 1.23E−8, which was equal to 0.05/Ne, where Ne is the effective number of independent tests [52]. To interpret GWAS results, significantly associated SNPs for each trait were first grouped into one locus in which two consecutive SNPs were less than 20 Kb, and in which at least two significant SNPs existed for each locus. The adjacent loci were further merged into a single locus if any significant SNPs between adjacent loci were in LD (*r*^2^ ≥ 0.2). To reduce the probability of missing causal genes in some narrow QTL intervals due to minor QTL, another 25 Kb was extended to both sides for those QTL with intervals less than 50 Kb and significance of the peak SNPs smaller than 100 times cutoff value (i.e., 1.23E−10). Ultimately, the significant loci were treated as sQTL, the peak SNP defined the significance of the sQTL, and the extended region of significant SNPs were defined as the sQTL interval.

### IBD-based GWAS (hGWAS)

The IBD mosaic map of all CUBIC lines was collapsed into 27,005 bins based on all identified recombination break points. In each bin, there was only 1 IBD state for a given line but 16~24 IBD states available across all lines. Treating each bin as 1 variable, the hGWAS was performed via the “JLM” script described previously [53]. In hGWAS, the mixed linear model was built and the restricted maximum likelihood (REML) was used to test the significance of each bin, by treating bin and polygenic effects as random effects and the top 10 PCs as fixed effects. The covariance structure of the polygenic effects was inferred with a bin-based kinship matrix, which was calculated using the method raised by [54] through reformatting bins as the dummy variables. The general analytic differences for the present population to previous study [53] included 2 aspects: (1) the fixed effect, the previous method (JLM) intended to jointly analyze the data with 10 independent populations; thus, a categorical variable or design matrix was included as fixed effect to indicate sample-population relationships; instead, the populations structure of CUBIC was inferred using PCA, which was fitted as fixed effects in hGWAS model (similarly what we do in sGWAS); (2) random effect, it was modeled by a kinship matrix under linear mixed model framework. Previously the kinship matrix was simply calculated by genome-wide SNP data, instead for CUBIC population, we inferred it using the whole-genome bin marker data. We used a permutation test of 500 iterations to determine the threshold for the likelihood ratio test (LRT) scores for each trait [55]. The resulting LRT threshold ranged from 6.8 to 7.4 (*α* = 0.05); for simplicity, we chose the average LRT of 7.1 as the overall cutoff for all traits. Following the previous procedures, significant bins were merged into a locus (or hQTL) with nearby positions (≤ 1 Mb) or located within ≤ 5 bins; the interval of each hQTL was defined as the physical position range delimited by the significant bin [53].

### GWAS simulation

To test and interpret the difference between the 2 GWAS methods, we simulated 3 possible QTL architecture scenarios using the CUBIC population, using biallelic quantitative trait nucleotide (QTN) expression as an SNP marker. These were as follows: (1) biallelic QTL, expressed as a single QTN at a given QTL; (2) 4-allelic QTL, attributed to 2 incompletely linked QTN at a given QTL; and (3) 9-allelic QTL, attributed to 3 incompletely linked QTN at a given QTL. We randomly selected 30 bins on chromosome 1 and set 10 bins as harboring single QTN for each bin; another 10 bins were assigned 2 QTNs per bin; and 3 QTNs assigned to each bin for the last 10 bins. We added a series of modest additive effects to QTNs, following the geometric distribution of *a*^*n*^ (*a* = 0.95), to simulate the polygenic nature of the complex traits [56]. The narrow-sense heritability (*h*^2^) and phenotypic variance (*V*_p_) were defined as 0.8 and 1 for the target trait, respectively. The simulated phenotype of each line was obtained by adding the sum of additive values to a residual error, following a normal distribution with 0 mean and variance of 0.2 (i.e., *h*^2^ = 0.8). The 2 GWAS methods described above were used to detect QTL in this simulation, which was replicated 1000 times to evaluate the statistical power and FDR for each QTL scenario.

### IBD-based QTL refinement by a linkage-like analysis

The major QTLs (those simultaneously detected by both GWAS methods with > 10% variance explained for the trait under study) were validated and refined. The major QTL region was defined as the union of the sQTL and hQTL intervals. The progeny lines were categorized into parental IBD groups according to the peak bin. Inferred from the simulation analysis that compared the statistical power of sGWAS and hGWAS, it may be a fact that multiple parents may share the same QTL allele. Thus, identifying or approximating the true QTL allele can reduce the model complexity (decrease the degree of freedom), which should increase the statistical power. But note that the IBD clustering for a QTL has to be phenotype dependent, which can reduce the impact of random variants on the clustering. Thus, for a QTL, the parental IBD groups were collapsed into phenotypically distinguishable clusters based on multiple comparisons of phenotype between the parent IBD groups (*P* < 0.05) for each trait. These IBD clusters, called functional alleles, reflected potentially true alleles dependent on specific traits, thus expecting to more closely predict trait variation than the parent IBD, independent of traits. At the QTL region, the inferred allelic types were projected onto all progeny lines, and the allelic combinations at the QTL region were re-clustered for the whole population into several major haplotypes. In this way, the pairwise comparison of phenotype between inferred haplotypes (as determined using the Student’s *t* test, *P* < 0.01) enabled the verification of the QTL credibility and further narrowed the QTL interval, in a manner similar to the traditional bi-parental fine-mapping procedure [57].

For fine mapping major GWAS loci, SNP and InDel polymorphisms located in the QTL interval were used to enable identification of the gene and the causal variant responsible for the GWAS identified locus. To help determine the likely functional genes, we ranked all genes via functional annotation (predicted by ensemble VEP program [58]) of polymorphisms located within the QTL interval, based on the generic feature format file version 3 (gff3) in maize following the listed procedure [59].

### Identification of epistasis in trait variance

Variants were filtered for frequency (MAF ≥ 2%) and linkage disequilibrium (*r*^2^ < 0.5 within each window of 50 Kb, with a step of five SNPs) before testing for epistasis. For each trait, the plink program [60] was employed in fast epistasis analysis using the supported boost test [61] with --fast-epistasis. Pairs of loci with *P* value > 6E−16 (roughly 0.01/*N*_*e*_^2^, where *N*_*e*_ is 4,057,944, the independent number of SNPs estimated by Genetic type 1 Error Calculator (version 0.2) [52]) were first removed, and only the most significant ones per pair were retained for those loci that interacted with multiple other loci within 100 Kb. The *P* value and corresponding variance explained for each putative epistatic pair were further adjusted by linear regression with controlling of population structure and additive effect, and those with adjusted *P* values < 1E−12 were kept as instances of significant epistasis. To estimate the trait variance jointly explained by epistasis, the population structure and additive effects for all remaining variants were first regressed out, and the residuals for each trait were further regressed against all interacting items. An extended interval of 50 Kb for interacting variants was used to determine the co-occurrence of identified epiQTL with previously identified networks.

### RNA sequencing, eQTL mapping, and epQTL analysis

A subset of 391 progenies were randomly selected from the CUBIC population for RNA sequencing. These lines and the founder parents were grown at the Hainan field station in the winter of 2016. At the V9 stage (the stage with the fastest leaf tissue growth), total RNA was extracted from the tissue of the 11th leaf, collected from the pool of 3 plants of similar growth status per line, following the standard protocol of Quick RNA isolation Kit, #0416-50 (Huayueyang Biotechnology CO., LTD. Beijing, China). A library with insert sizes ranging from 200 to 500 bp was prepared using the commercial Illumina library preparation kits (TruSeq Stranded mRNA LT-SetA. RS-122-1201). A 150-bp paired-end Illumina sequencing was performed using the HiSeq X-Ten protocols. Each sample had on average ~ 20 million raw reads. The reads with low sequencing quality and sequencing adapter were removed using the software Trimmomatic-0.36 [40]. The paired-end reads were mapped onto the B73 AGPv3.25 reference using the software STAR [62] with a maximum intron size of 50,000 bp; only those uniquely mapped reads were used to quantify gene expression levels using the HTSeq [63]. The expression data for each gene was normalized using the software DESeq2 [64] before the subsequent analyses.

Only genes expressed in more than 60% of the lines were retained in eQTL mapping. Top ten PEER [65] factors, together with the top ten genotypic PCs, were utilized to account for covariates. The software EMMAX [66] was used in eQTL mapping, taking 1.89E−8 as a significant standard. The co-localization of pQTL and eQTL was measured with the evidence that the peak variant of pQTL was included in significant SNPs from eQTL. Those loci affecting trait variance by regulating those genes outside the pQTL region and not due to LD (> 10 M) were considered candidates with trans-effect. The OSCA program [32] was used to perform expression-phenotype associations (epQTL) under a linear mixed model, with 1E−3 as a significant threshold.

### Cross-population analysis

The mapping on a maize association mapping panel (AMP) containing 508 unrelated inbred lines [67] was used to cross-validate and narrow down the candidate list. Since the mapping results of AMP were based on the B73 v4 genome, we first extracted the candidate genes located in 776 QTL in the present CUBIC population and transformed the B73 v3 gene ID into B73 v4 gene ID. All CUBIC QTLs were applied to candidate association mapping in AMP along corresponding traits and considered as significant when *P* value < 1E−4. Meanwhile, the cross-validation in the maize association population makes the number of candidate genes greatly reduced.

### Metabolic analysis

A metabolic profile was conducted to help interpret how genes identified in this study may be involved in regulating the phenotype. From the 391 lines used in RNA-seq, a subset was selected based on the phenotype and inferred allelic types in the specific QTL under study. For the major QTL identified for ELW on chromosome 4, a total of 71 lines were chosen based on the genotype of peak SNP, with 35 vs. 36 for different alleles of the 1 bp deletion in *ZmGalOx1*. These lines and the founder parents were grown at the Hainan field station in the winter of 2016. At the V9 stage, we collected tissue from the 10th leaf, collected from 3 plants per genotype, for metabolic analysis. The 7th leaf of the CRISPR-Cas9 edited T0 plants was also collected at the V9 stage from plants grown in the greenhouse of the China National Seed Group Co., LTD. (Wuhan, China), in the autumn of 2017. The primary metabolites were extracted from maize leaves following the procedures described in detail in previous studies [68, 69]. In brief, 50 mg of lyophilized leaf powder was extracted with 1 ml of methanol: methyl-t-butyl-ether (MTBE) solution (1: 3 v/v). A 300-μl aliquot from the lower polar phase was taken and dried in a vacuum. The dried residue was derivatized with *N*-methyl-*N*-(trimethylsilyl) trifluoroacetamide (MSTFA) and further analyzed by GC-MS (7890A-5975C, Agilent, Santa Clara, USA) following the protocol described in previous studies [70, 71].

Hormones were extracted from maize leaves according to the protocols published previously [72], and the hormone compounds were separated by reversed-phase ultra-fast LC (Shimadzu, Kyoto, Japan). These were detected by electrospray ion source of a tandem triple quadrupole MS analyzer (API4000, AB SCIEX, Singapore) and quantified in multiple reaction monitoring (MRM) mode using optimized MS/MS conditions. The MS conditions were as follows: source, Turbo IonSpray; ion polarity, negative; IonSpray voltage, − 4500 V; source temperature, 550 °C; gas, nitrogen; curtain gas, 30 psi; nebulizing gas (GS1), 55 psi; collision gas (GS2), 55 psi; scan type, MRM; Q1 resolution: unit; and Q3 resolution: unit. The Analyst 1.5.2 software (AB SCIEX, Foster City, CA, USA) was used to control the instrument and to acquire and process all MS data. Among all samples, ten samples were tested twice for technical replications. The Pearson correlations between two replications indicated the high quality of metabolic data (*r* = 0.9927 ± 0.0083).

### CRISPR/Cas9 editing experiment

In order to confirm that *ZmGalOx1* is the causal gene for the ear leaf width QTL identified via GWAS on chromosome 4, we edited the gene sequence using the CRISPR-Cas9 system. Two guide RNAs (gRNAs) targeting the 14th exon of *ZmGalOx1* were designed using the CRISPR-P [73]. The 2 gRNAs were integrated into a highly efficient vector [74] and transformed into immature embryos of ZZC01 by Agrobacterium infection by the China National Seed Group Co., LTD. A total of 55 T0 CRSPR-Cas9 edited seedlings were obtained (including positive and negative control plants), and DNA was extracted from each individual at the seedling stage. Primers were designed to amplify about 800 bp in the vicinity of (and including) the 2 gRNAs within the gene. The PCR products were used for Sanger sequencing to examine the variations along the sequence. By comparing the sequences with those of the unedited lines (ZZC01) using the BioEdit software [75], all sequence changes that could lead to amino acid changes were identified. For all 55 T0 plants, ear leaf widths in the R1 stage (after pollination) were measured for statistical analysis.

### Leaf cytological experiment

Lines with the most extreme ear leaf width, and having either the allelic type with or without the 1-bp insertion in *ZmGalOx1*, were planted in Wuhan in the summer of 2017. Ear leaves were collected in the R1 stage to prepare for the cytological experiment. In the autumn of 2017, the ear leaves of CRISPR/Cas9 edited plants (including positive and negative control plants) were also collected at the R1 stage from greenhouse-grown plants. The abaxial leaf epidermis tissue was removed 20 cm from the leaf tip and affixed to a microscope slide. Images were taken under × 10 field in an Olympus DP72 compound microscope with × 10 eyepiece. Counting in a direction perpendicular to the leaf vein, the number of cells per field was counted by the Image-Pro Plus software [76] to determine the average width of a single cell. The total number of cells along the leaf from left to right was calculated as leaf width divided by the average single cell width. For each plant group, 12 plants were investigated for cell size and cell number with 3 leaves per plant. The 3 values were averaged for each plant. Student’s *t* test was used to test the significance of difference between the positively and negatively edited plants and between different *ZmGalOx1* genotypic plants (*P* < 0.01).

## Supplementary information

**Additional file 1.** Information of 24 elite maize inbred founders.

**Additional file 2.** Whole-genome sequencing statistics and population structure.

**Additional file 3.** Supplementary notes, Figures S1–S19 and Tables S1–S5.

**Additional file 4.** Information of 23 maize agronomic traits investigated in CUBIC population.

**Additional file 5.** Summary of QTL via sGWAS.

**Additional file 6.** Bin information across whole genome.

**Additional file 7.** Summary of QTL via hGWAS.

**Additional file 8.** List of significant epistatic QTLs.

**Additional file 9.** Information of 14 genes located in the ELW QTL on chromosome 4.

**Additional file 10.** Identification functional candidates co-mapped by sQTL by eQTL mapping.

**Additional file 11.** List of sQTL showing trans-eQTL effects.

**Additional file 12.** List of expression-phenotype associations (epQTL).

**Additional file 13.** Narrowing QTL by cross-population analysis.

**Additional file 14.** Consensus map integrated by 10 recombinant inbred line (RIL) populations.

## Data Availability

Raw whole-genome sequencing and RNA sequencing reads of CUBIC population are available on NCBI with Bioproject accession number PRJNA597703 (https://www.ncbi.nlm.nih.gov/bioproject/PRJNA597703) [77] and also have been deposited in the Genome Sequence Archive [78] in BIG Data Center [79] under accession codes “CRA000171” [80] and “CRA001241” [81], respectively. The script for the construction of the IBD mosaic map can be accessed at GitHub [82].
