# Supplementary material for: CUBIC: an atlas of genetic architecture promises directed maize improvement
Source: Genome Biol. 2020 Jan 24;21:20. doi: 10.1186/s13059-020-1930-x (PMC6979394; doi:10.1186/s13059-020-1930-x)
Supplement: Supplementary file 3 — Additional file 3. Supplementary notes, Figures S1–S19 and Tables S1–S5. [file 13059_2020_1930_MOESM3_ESM.pdf]

Supplementary Information for

CUBIC: an atlas of genetic architecture promises directed maize improvement

This file includes:

Supplementary Notes.

Supplementary Figures

Fig. S1. Pipeline for variant calling, genotyping and imputation.

Fig. S2. Phenotyping locations.

Fig. S3. Population structure and LD decay of CUBIC panel.

Fig. S4. Manhattan plots for 23 agronomic traits based on sGWAS.

Fig. S5. Mosaic map of identity-by-descent (IBD) for 24 founder parents.

Fig. S6. IBD based recombination pattern in the progeny across the genome.

Fig. S7. Manhattan plots for 23 agronomic traits based on hGWAS.

Fig. S8. Genomic modeling for inbred improvement.

Fig. S9. Significant epistasis in trait variance.

Fig. S10. Comparisons of epiQTLs and known gene-gene networks.

Fig. S11. Enrichment of intergenic QTL on the MNase hyposensitive (HS) proximal regions.

Fig. S12. Inference of functional allelic types across parent IBD.

Fig. S13. Cytological experiment for 59 well-selected mature leaves in the CUBIC population based on the genotype.

Fig. S14. The possible metabolic pathway involved in ZmGalOx1.

Fig. S15. Identification of functional genes by cross-population analysis and cross-omics mapping.

Fig. S16. Cases for eQTL mapping in identification of functional genes.

Fig. S17. Novel functional candidates revealed by epQTL mapping.

Fig. S18. Global IBD proportion of 24 parents in CUBIC population.

Fig. S19. High reliability of HMM method revealed by simulation analysis.

Supplementary Tables

Table S1. The phenotype statistics for the 23 agronomic traits measured in this study.

Table S2. Summary of genetic contributions to trait variance.

Table S3. Known metabolites that are significantly correlated with ear leaf width.

Table S4. The information of *ZmGalOx1* type-I polymorphisms.

Table S5. The allelic variations of 24 parents at the *ZmGalOx1* type-I polymorphisms.

Supplementary Notes

Creation of desirable inbred lines by genomic modeling

Through the integration of QTL with relatively large phenotypic effects, this study provides a practical opportunity for inbred line improvement and could be further applied to the precise customization of elite parental lines for hybrid breeding. Moving beyond the potential application of individual QTL, we now demonstrate the value of the ensemble model in pyramid breeding. To simplify the issue, we chose EW, DTA, and PH as representative of yield, flowering time, and plant architecture, respectively, which are main target traits in maize improvement programs.

The QTL identified for EW also show various effects on DTA and PH (**Fig. S8a**). This is readily understandable because ear weight is affected by dynamic and complex combinations of traits including DTA and PH (**Fig. S8b**). During offspring selection, we had already produced lines with higher (or comparable) yield, earlier flowering, and reduced plant height as compared with all parents (**Fig. S8b**). However, not all favorable alleles for EW QTL were present in any offspring line, indicating that there is still room for yield improvement in future generations. By integrating the absent favorable alleles, the whole population displayed another 27.6g (+30.3%) of growth potential, on average, for EW, with little change (-1.3%) in flowering time but a relatively large increase (+11.1%) in plant height (**Fig. S8c**). Genomic modeling could provide a path for breeders to follow towards breeding optimization of all desirable alleles for all required traits. Considering multiple phenotypes together is essential and more productive than a simpler approach, since a large fraction of the genetic basis of complex traits is shared, even for those non-correlated ($p>0.05$) traits (**Fig. S8de**). The results of the present project allow us to estimate the greatest yield potential for any given genotype, with limited alteration (or simultaneous optimization) of other traits.

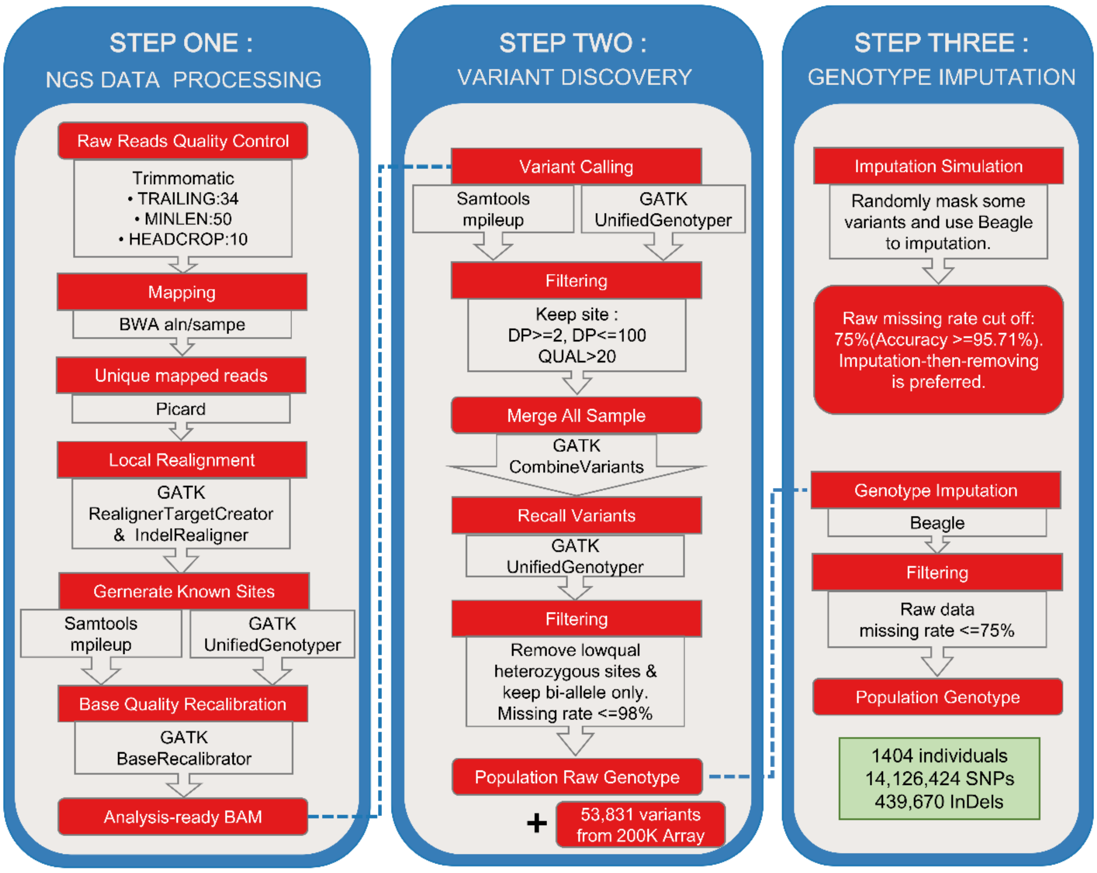

Fig. S1. Pipeline for variant calling, genotyping and imputation. At each stage, the boxes colored in red represent corresponding procedures, followed by descriptions and specific parameters in colorless boxes. For genotype imputation (step three), the second colored box represents the simulated standard adopted, and the box in green presents the final variant statistics.

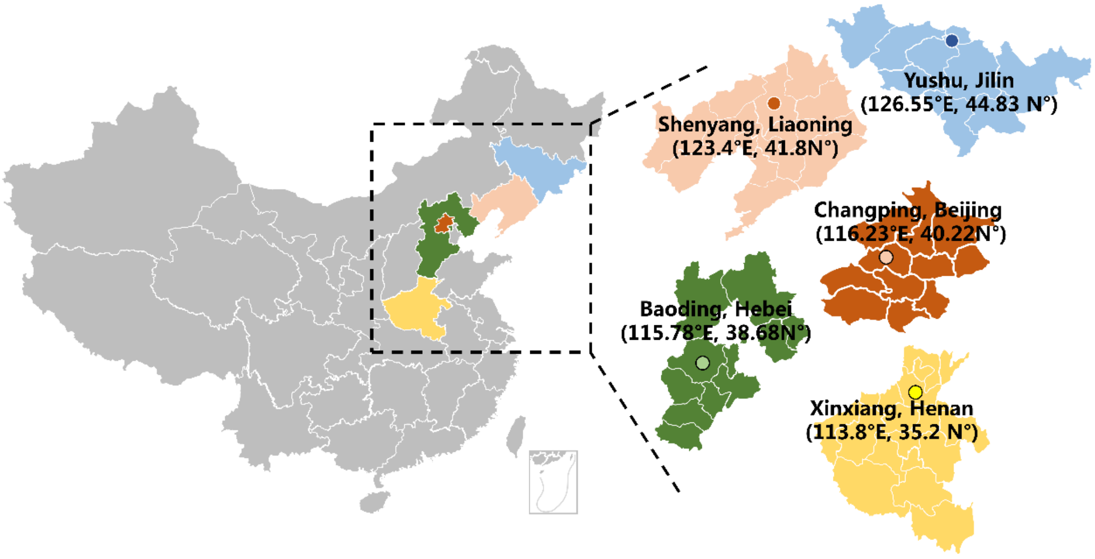

Fig. S2. Phenotyping locations. Phenotyping trials were carried out in the major maize producing areas in China and where the 24 elite founders are the most adapted**.**

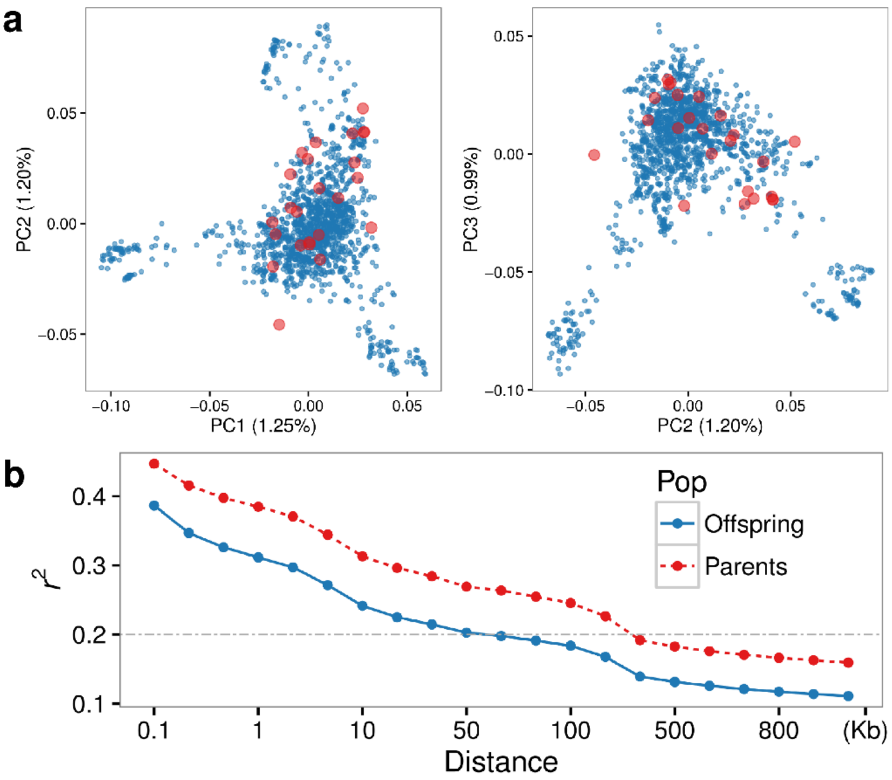

Fig. S3. Population structure and LD decay of CUBIC panel. (a) Principal component analysis reveals a weak population stratification, and a higher diversity of offspring (blue) compared to 24 founders (red). **(b)** Comparison of LD decay between founder and offspring panels. The x-axis represents the distances of adjacent SNPs in Kb, and the y-axis is the average r-square along various interval distances.

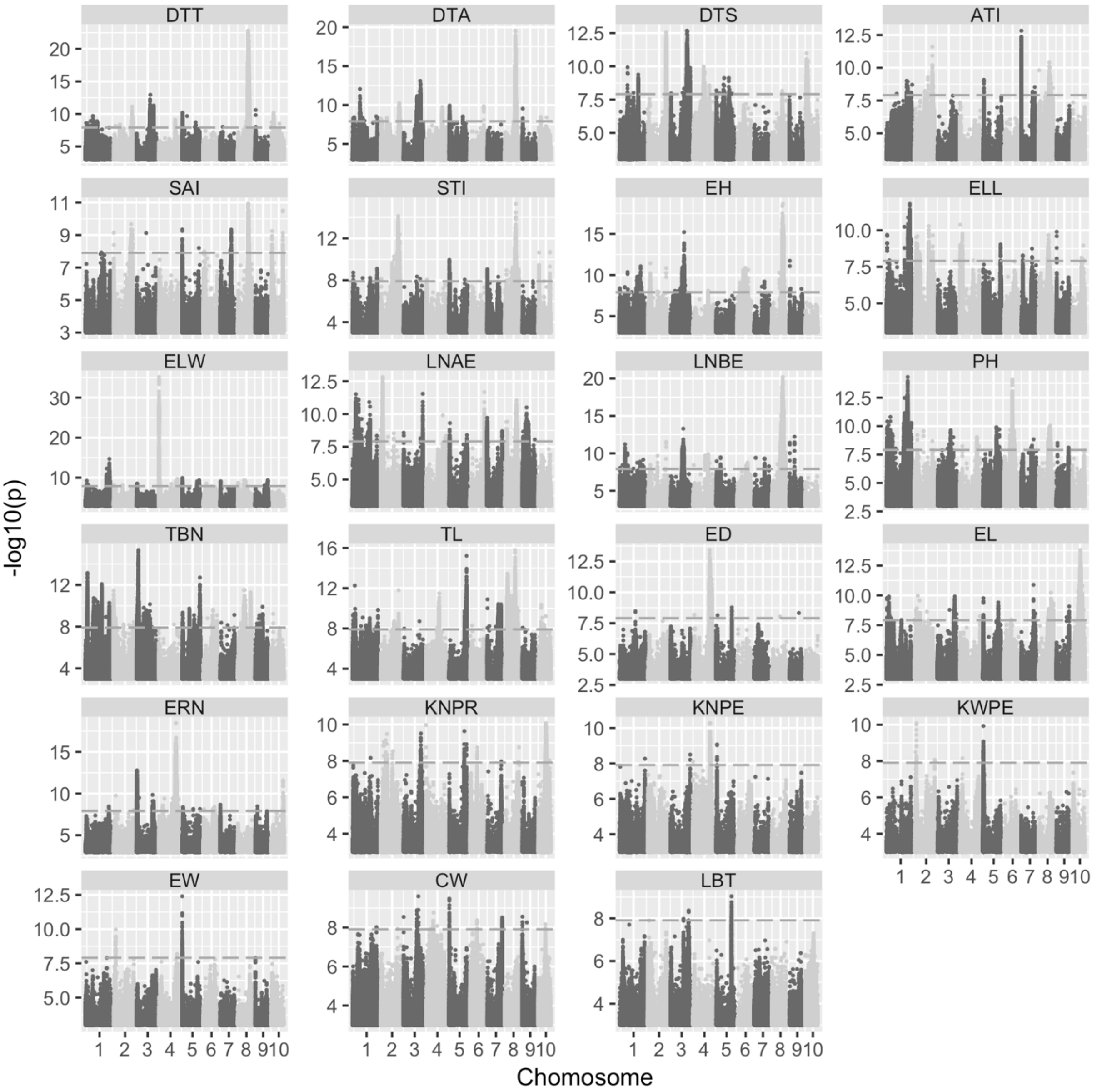

Fig. S4. Manhattan plots for 23 agronomic traits based on sGWAS. The horizontal dashed line in each plot indicates the *P* value threshold ($P<1.23\text{E-}8$) for declaring the significance of associations for SNP polymorphisms with each trait, based on the Bonferroni method, i.e., $P<0.05/Ne$, where *Ne* is the effective number of independent tests.

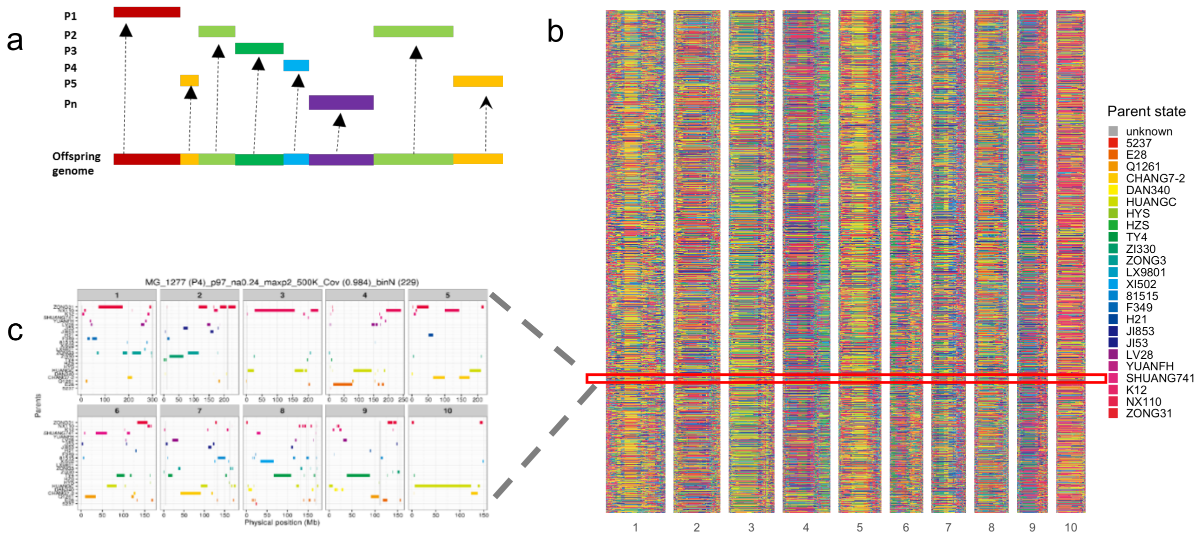

Fig. S5. Mosaic map of identity-by-descent (IBD) for 24 founder parents. (a) Example schematic of an offspring line showing regions derive from multiple parents. **(b)** Genome-wide IBD pattern for all offspring lines. **(c)** A zoom-in graph of IBD pattern of one specific line. The colors indicate the IBD states descended from specific founder parents, as listed to the left of (b). The segment is assigned to the parent with the top probability, only if it is larger than 2 folds of expected value by chance (1/24), otherwise it was considered an unknown state.

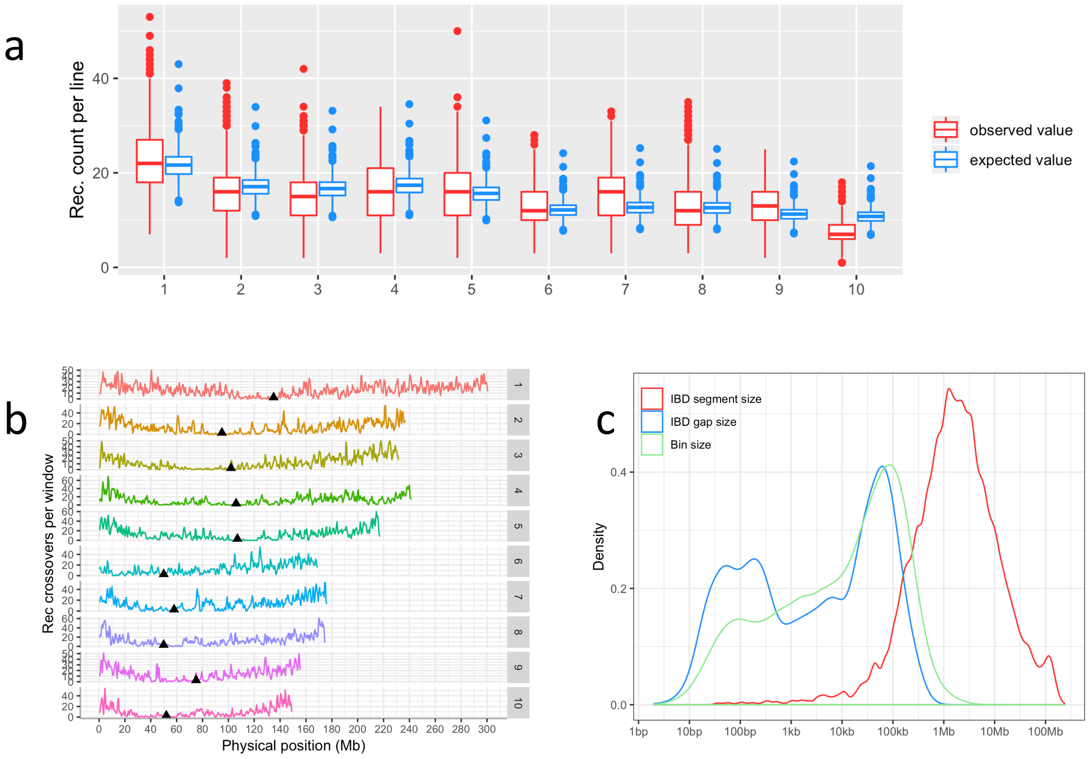

Fig. S6. IBD-based recombination pattern in the progeny across the genome. (a) Recombination events for 10 chromosomes. The red box indicates the observed number of recombinations per line, and the blue box is the expected value, obtained by assuming recombination was evenly distributed across the genome, followed by multiplying the sum of recombination per line with the proportion of total genomic physical size for each chromosome. The colored triangles below the box indicate the significant difference between real value and expected value, by two-tail *t* test (P<0.01); red triangle indicates the observed recombination was more than expected value, but lower than expected in blue triangles. **(b)** The recombination pattern along the maize chromosomes. Each estimated point indicates the mean value of recombination jointly in a 1-Mb sliding window across the 1,404 progeny lines. **(c)** The resolution of the IBD mosaic map for genetic mapping.

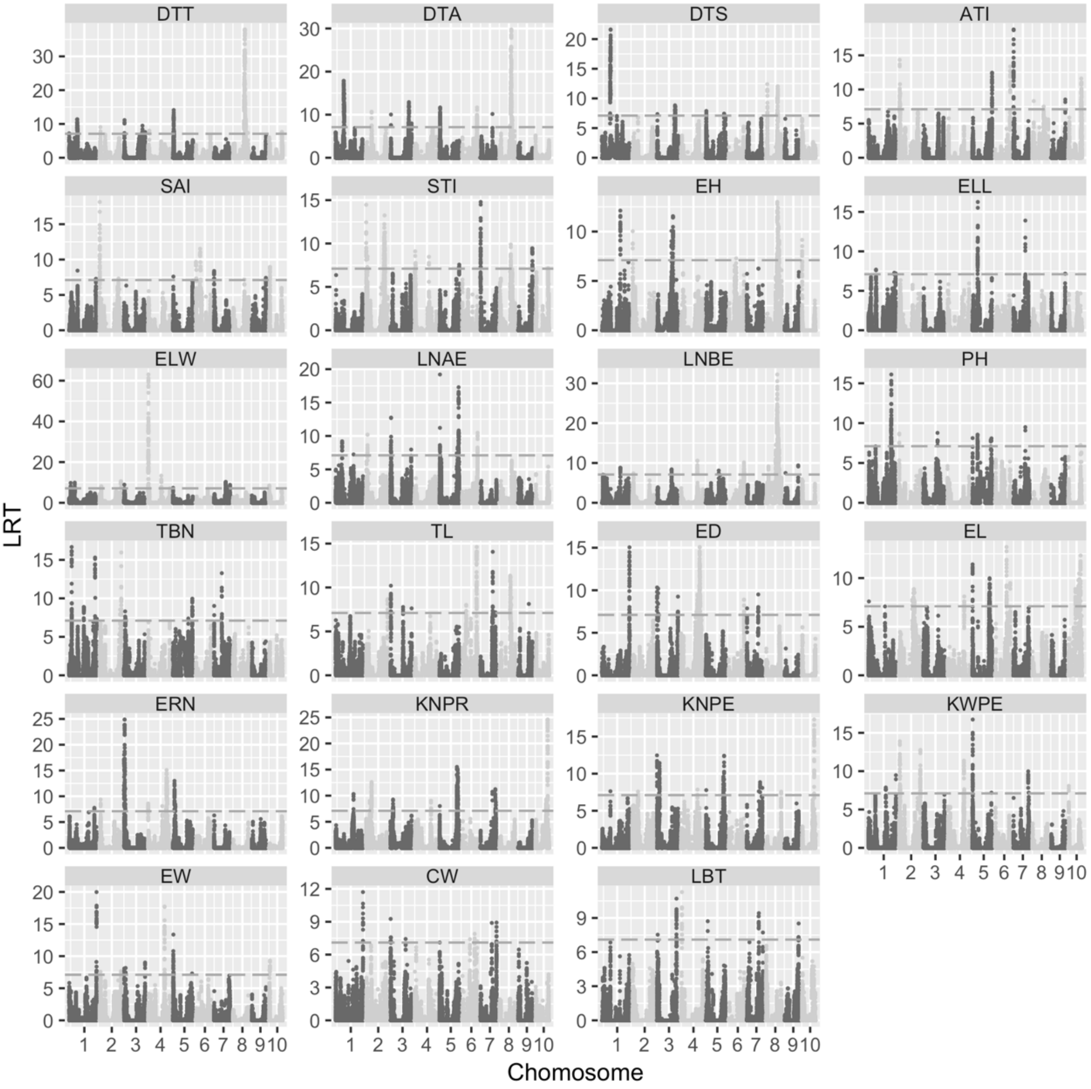

Fig. S7. Manhattan plots for 23 agronomic traits based on hGWAS. The horizontal dashed lines in each plot indicates the likelihood ratio (LRT) threshold declaring the significance of association of each bin across the genome. The LRT threshold was determined by 500 permutations for each trait, which varied from 6.8 to 7.4. In the present study, we chose the average of 7.1 as the unique cutoff of hGWAS for all traits.

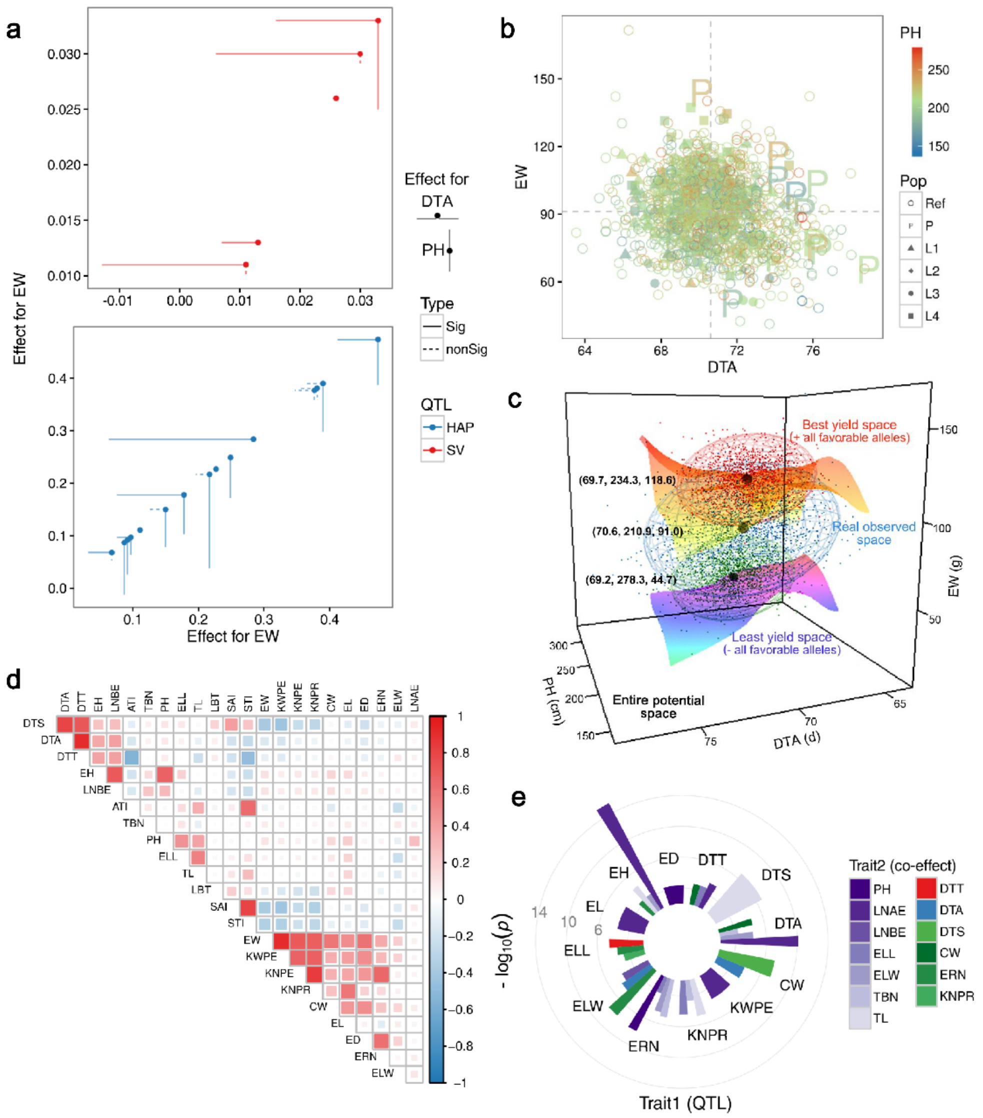

Fig. S8. Genomic modeling for inbred improvement. (a) QTL for EW showed a range of effects on DTA and PH. Different colored points represent QTL identified under different models (SV- and HAP- GWAS), with x-axis or y-axis representing the effect size on EW. The line extending from each point indicates its additional effects on DTA (horizontal) and PH (vertical); the length represents corresponding effect size; and direction represents either the co-directional effect compared with EW (rightward or upward; phenotypic value was positively correlated) or opposite effect (leftward or downward; trait was negatively correlated). Solid lines indicate significance at $P<0.05$ and dashed lines indicates lower levels of significance for the effect of EW QTL on other traits. **(b)** Changes in EW in concert with DTA and PH, observed in the present population. **(c)** The observed and predicted space of EW along axes for DTA and PH for the whole population. The best (red points and red-yellow surface) and worst (green points, blue-purple surface) performances were predicted by integrating and removing all favorable allele/haplotypes for EW within each line, respectively, compared to the observed trait value (blue points) at specific genotypes. The three large black points were mean performance of DTA, PH, and EW in the corresponding spaces. **(d)**

Measuring phenotypic correlations using trait values. **(e)** Measuring cross-phenotypic correlations using genetic sharing. For each trait, all corresponding QTLs (Trait 1 (QTL), represented by peak trait value variants) identified by GWAS (SV-MLM) were tested if they affected other non-correlated (P>0.05) traits (Trait2 (co-effect)) in this study. The circular axis represented the significance ($-\log_{10}$(p-value), increasing moving outwards) of the effects on Trait2 from QTLs from Trait1, using linear regression by controlling population structure, additive effects and the phenotypes of Trait1.

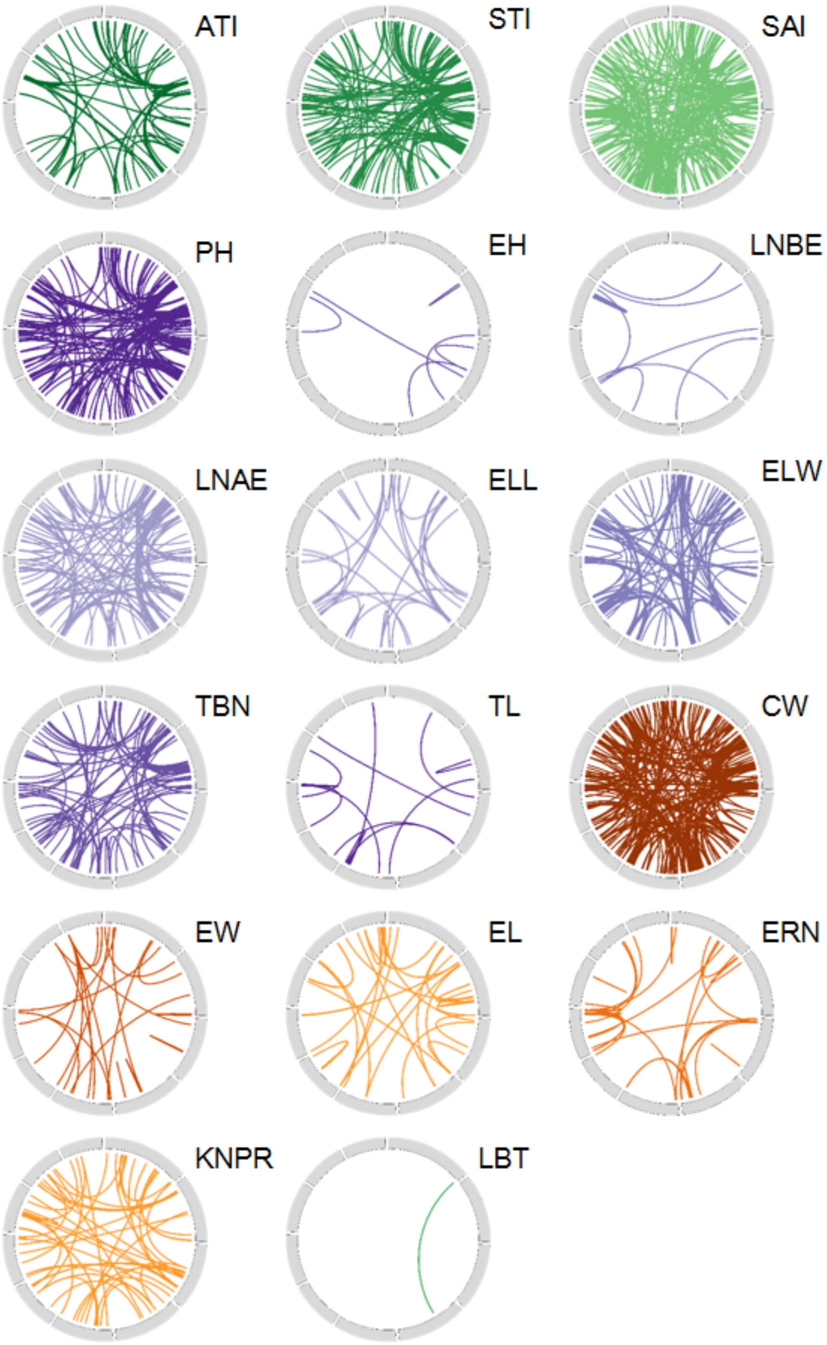

Fig. S9. Significant epistasis in trait variance. Continued from **Fig. 3a**. Each circle represents a trait (with arbitrarily selected color), and each line links an epistatic pair of loci, on chromosomes 1-10 within each circle (listed in order, clockwise from top indicating chromosome 1). Traits without significant interactions are not indicated. Full names of traits are referred in Methods section.

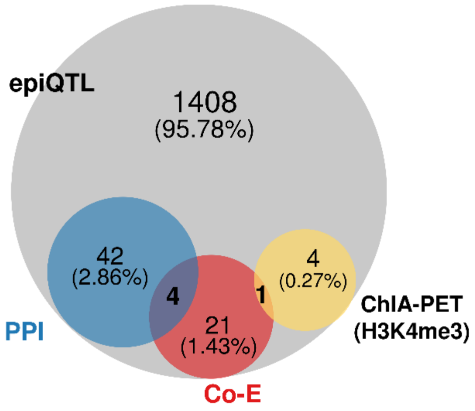

Fig. S10. Comparisons of epiQTLs and known gene-gene networks. Circles and numbers within each circle include total number of epiQTL found (gray circle) and co-occurrence of epiQTLs and interacting genes found by other methods. Blue circle, PPI: protein-protein-interactions [25]; Red circle, Co-E: co-expression relationships in protein and transcript levels [26]; Yellow circle, ChiA-PET: the interacting genome architecture from experiments on H3K4me3 modifications [27].

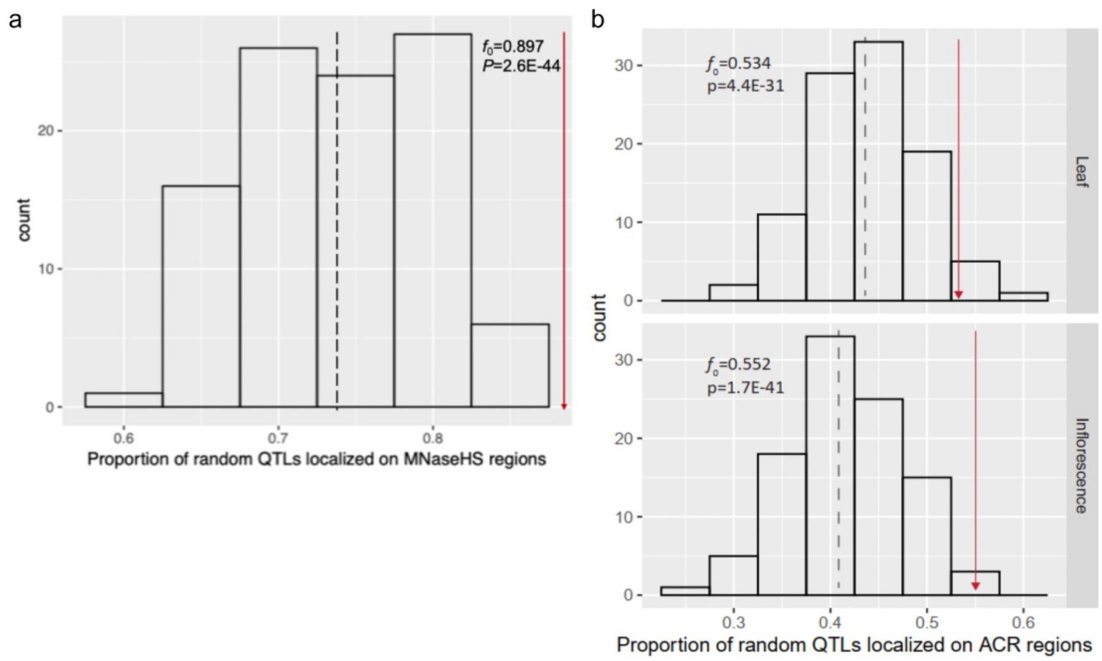

Fig. S11. Enrichment of intergenic QTL on the potentially functional elements. The plot shows the distribution of observed and expected QTL-MNaseHS (MNase hyposensitive) co-localization **(a)** and QTL-ACR (accessible chromatin regions) co-localization **(b)**. The expected distributions were obtained by randomly drawing the same number of intergenic QTL regions and comparing them with the MNaseHS and ACR regions identified in maize [28, 29]. The dashed black line indicates the mean of random colocalization proportion (f_e), and the solid red arrow indicates the observed colocalization proportion (f_o) in this study. The one-sample t test was used to test whether the f_o is derived from the distribution of f_e. The one-tail P-values indicate rejection of the null hypothesis and provides statistical proof that the observed value is significantly higher than the expected in this data set.

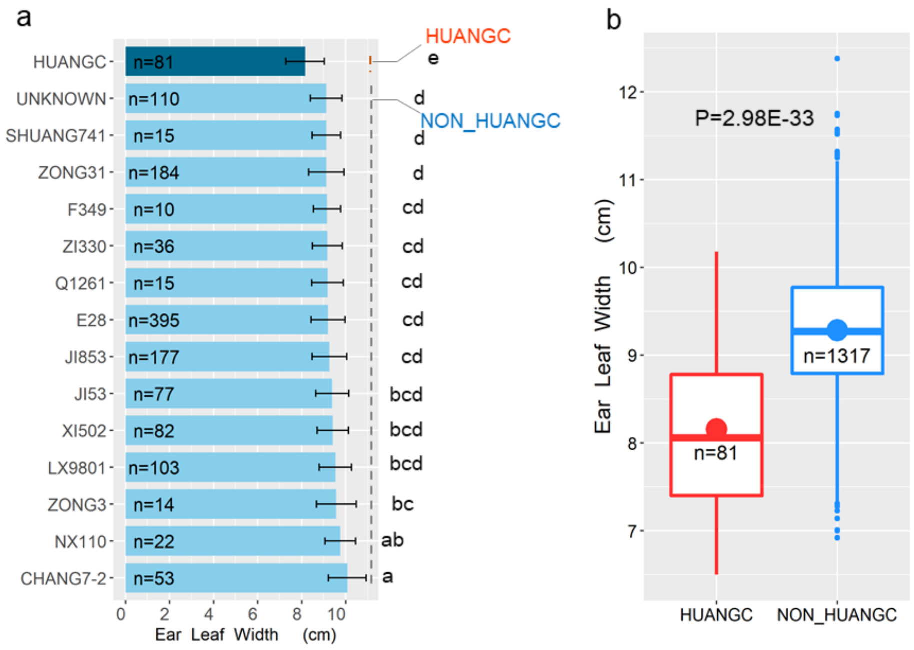

Fig. S12. Inference of functional allelic types across parent IBD groups. (a) The spectra of parent IBD effect in this QTL interval ($n \geq 10$). The bar and error line indicate the mean and standard deviation of ear leaf width (ELW) in each parent IBD group (and all unknown IBD bins). The number shown in the bar indicates the line count assigned to each IBD group. Multiple comparison significance is indicated by letters, based on the HUANGC functional allelic type, or all others (NON_HUANGC; which were not significantly different between them). **(b)** Genetic effect of functional allelic types. The horizontal line within the box indicates the median value; the bars of the box indicate the limits as 1.5 times the interquartile range from the box; the dots outside the bars indicate the most extreme data points or possible outliers. The number in the box is the number of lines assigned to each functional allelic type. The student's t test was used to evaluate the difference in ELW between allelic types.

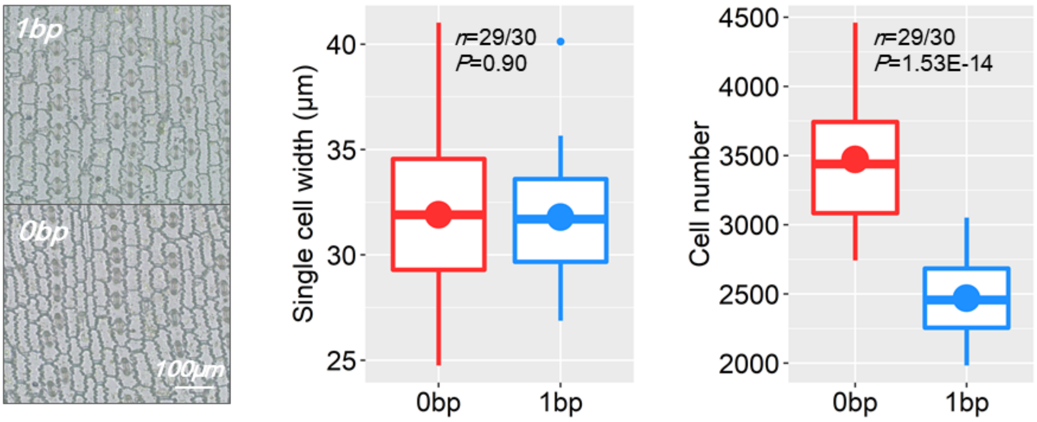

Fig. S13. Cytological experiment for 59 selected mature leaves in the CUBIC population based on the genotype. Left panel, epidermal cells in the abaxial leaf surface for the genotype with (top of panel) and without (bottom of panel) the 1bp insertion. Middle panel is the width of single cells for lines with (blue) and without (red) 1bp insertion, which showed no significant difference. Right panel, number of cells along the widest part of the leaf blade for plants with (blue) and without (red) the 1bp insertion, which showed a significant difference and is the cause of the size difference in the ear leaves.

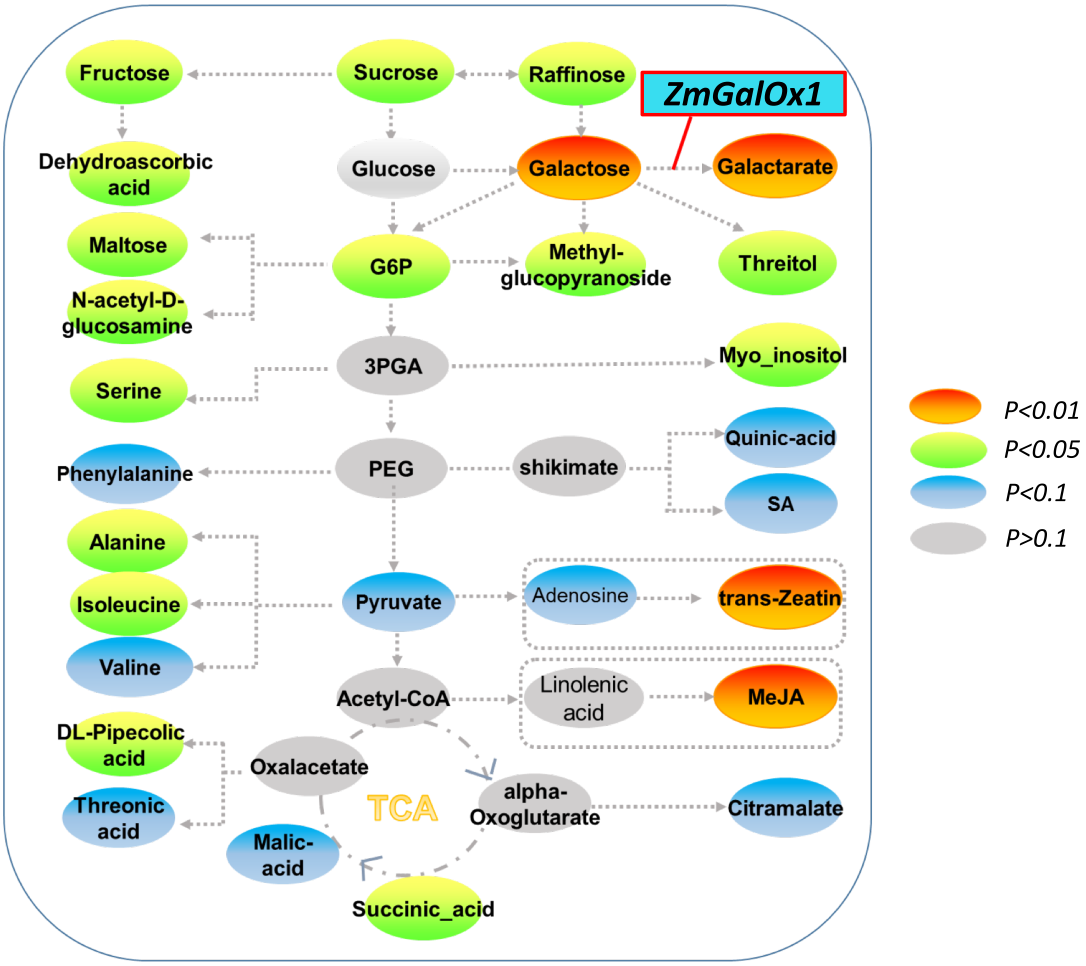

Fig. S14. The possible metabolic pathway involved in *ZmGalOx1*. Metabolites which were significantly correlated with ear leaf width both in the CUBIC progenies and genome edited lines were mainly involved in the glycolysis pathway, tricarboxylic acid cycle (TCA), and the synthesis of some plant hormones. The causal gene is marked in the blue filled rectangle. Orange, green, blue and grey ovals represent the metabolites which were present at a significance level below 0.01, 0.05, 0.1 and larger than 0.1, respectively.

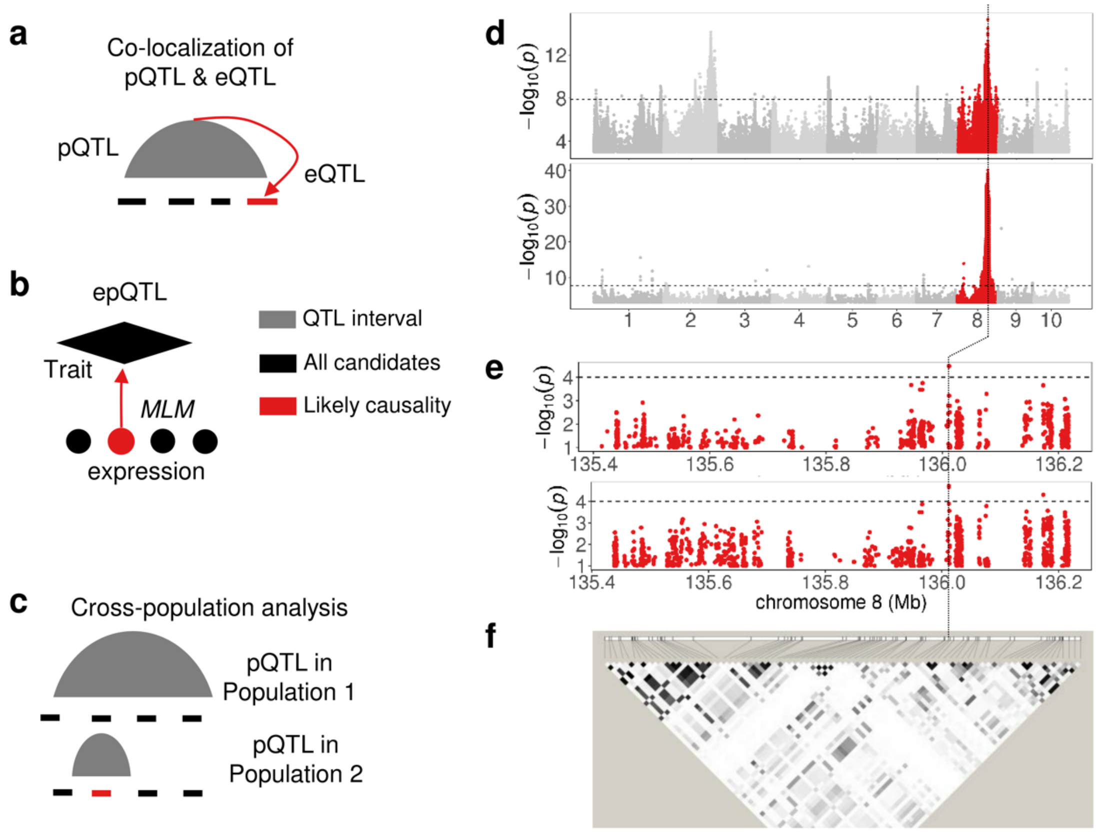

Fig. S15. Identification of functional genes by cross-population analysis and cross-omics mapping. (a) Narrowing down candidates by co-localization of eQTL and pQTL, that those candidates with expression simultaneously regulated by pQTL were likely functional. **(b)** The candidates whose expression significantly associated with trait variance were likely functional. **(c)** Narrowing down candidates by cross mapping in various populations, that the genes being identified significantly associated to same or proximity phenotypes had greater chance to be causal. **(d)** The co-localization of pQTL for trait STI (above panel) and eQTL for *RAP2* (AP2/EREBP transcription factor, GRMZM2G700665; below panel) at chromosome 8. **(e)** Taking the QTL region to perform candidate genes association analysis, *RAP2* was identified significantly associated with flowering time traits (DTT above and DTS below) in another un-related population. Thus the *RAP2* (marked as black dash) can be proposed as a high potential functional gene. **(f)** LD structure of candidate *RAP2*. The most significant variant was marked as black dash.

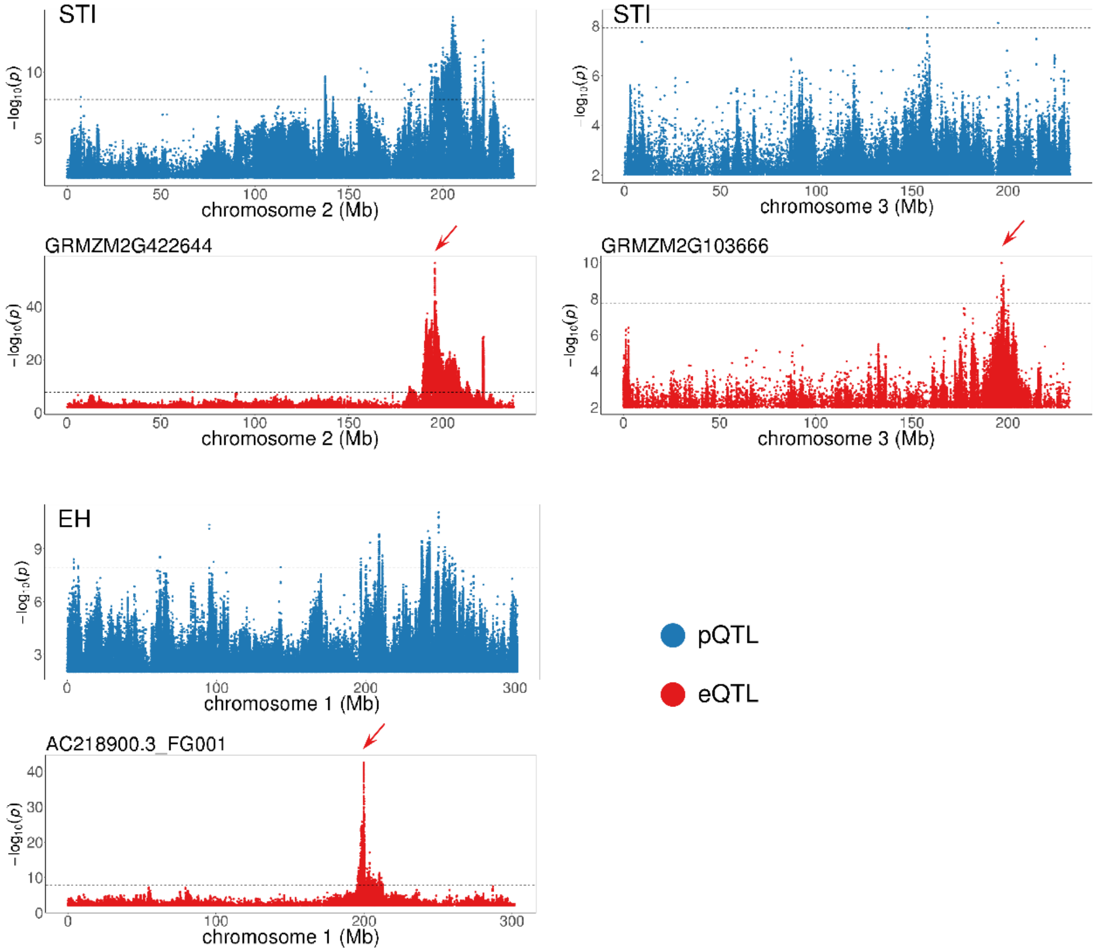

Fig. S16. Cases for co-localization of eQTL and pQTL in identifying functional genes. For each pair, the above Manhattan plot is for SNP-trait associations, and the below is for the gene who is located within the pQTL and whose expression is simultaneously regulated by pQTL.

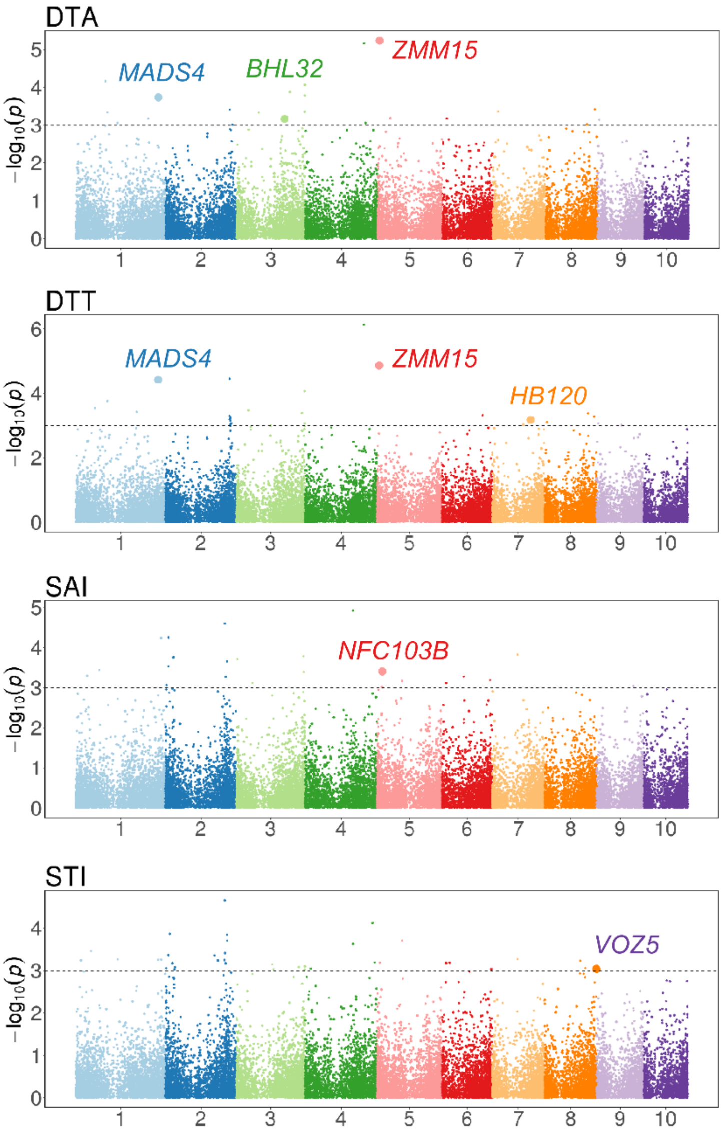

Fig. S17. Novel functional candidates revealed by epQTL mapping. Each panel is for one trait marked in top left, and the point measured by the significance between gene's expression and trait variance in association analysis under linear mixed model [32].

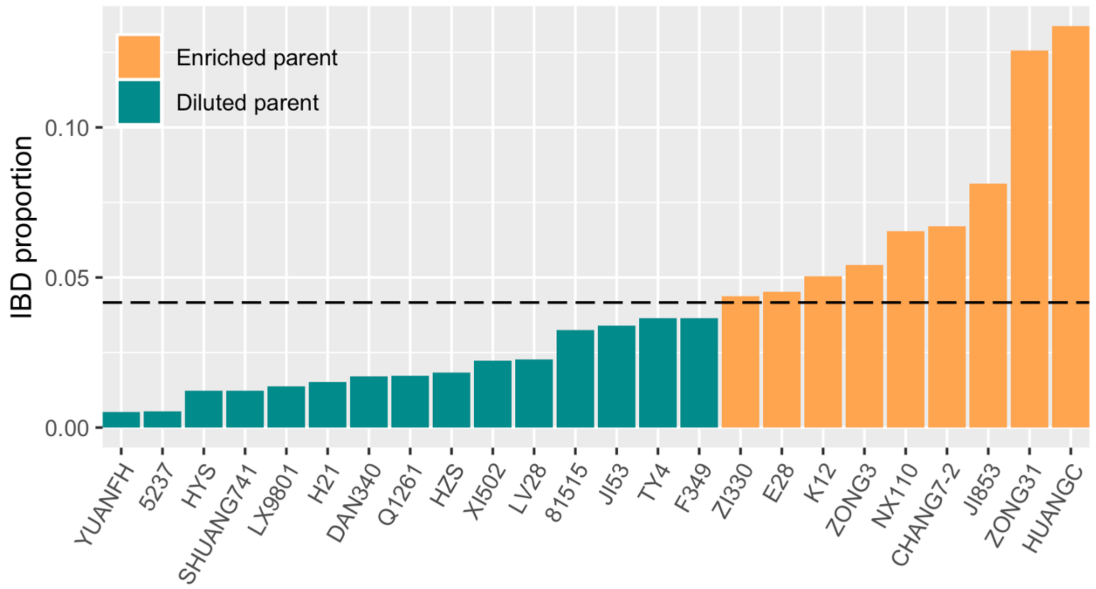

Fig. S18. Global IBD proportion of 24 parents in CUBIC population. The horizontal dashline indicates 1/24 as the expected IBD proportion assumed by chance. The parents with global IBD proportion higher than the expected value are designated as enriched parents, otherwise, as diluted parents.

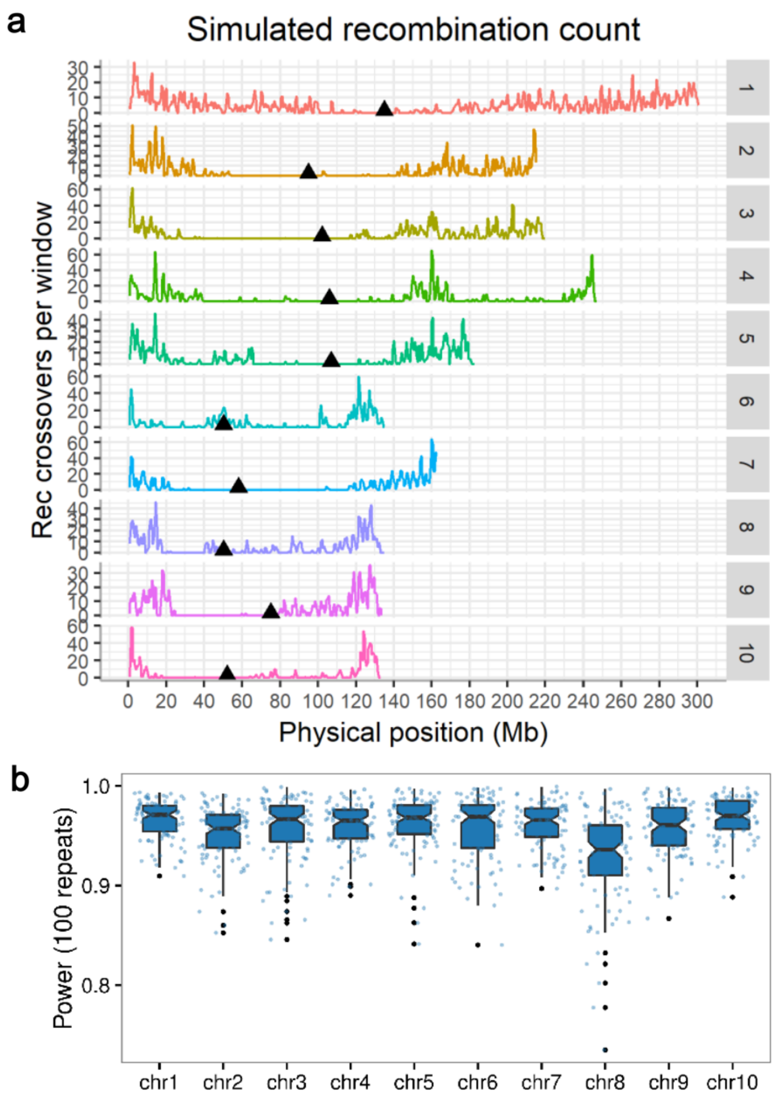

Fig. S19. High reliability of HMM method revealed by simulation analysis. (a) The recombination events from random simulated 100 progeny lines reflected a vivid landscape. **(b)** The HMM method showed a high power to correctly identify the founder origin of CUBIC dataset.

Table S1. The phenotype statistics for the 23 agronomic traits measured in this study. Mean, standard deviations, and range in phenotypic performance are listed. Trait abbreviations can be found in Online Methods.

Trait	Parent		Progeny		H^{2a}	Mean±SD(AMP)[b]
	Mean±SD	Range	Mean±SD	Range		
DTT	69.46 ± 3.23	64.33~76.52	67.43 ± 2.56	59.36~77.8	0.87	67.34±3.77
DTA	72.64 ± 2.96	68.51~78.92	70.64 ± 2.2	63.56~80.8	0.86	70.20±3.72
DTS	74.72 ± 2.89	70.78~81.09	73.43 ± 2.41	67.04~85.55	0.86	72.18±3.62
ATI	3.13±0.88	1.82-5.45	3.24±0.88	1.48-6.66	0.72	2.86±1.09
SAI	2.15±1.25	-0.58-4.42	2.74±1.27	-0.62-7.92	0.78	1.98±1.33
STI	5.29±1.56	2.52-8.02	5.98±1.75	1.18-11.91	0.82	4.84±1.56
EH	86.03 ± 9.66	67.43~100.79	83.72 ± 12.97	48.59~131.2	0.91	64.70±13.65
ELL	75.7 ± 5.73	66.68~90.78	78.35 ± 6.07	60.75~98.88	0.88	71.55±7.08
ELW	9.43 ± 0.59	8.31~10.74	9.22 ± 0.83	6.5~12.38	0.87	8.49±0.82
LNAE	6.34 ± 0.32	5.62~6.85	6.48 ± 0.53	4.88~8.37	0.83	n.a.
LNBE	8.33 ± 0.51	7.45~9.04	7.65 ± 0.72	5.44~10.88	0.85	n.a.
TBN	13.14 ± 3.86	6.91~20.04	12.07 ± 3.42	4.47~29	0.91	9.44±3.38
PH	201.69 ± 16.66	166.37~236.16	210.87 ± 20.11	139.1~283.25	0.92	173.15±21.58
TL	32.15 ± 3.25	25.33~38.53	33.14 ± 4.14	20.3~47.56	0.93	30.26±3.33
ED	4.21 ± 0.28	3.67~4.67	4.16 ± 0.25	3.14~5	0.75	3.70±0.32
EL	13.57 ± 1.79	9.24~17.24	13.38 ± 1.41	8.76~19.05	0.84	12.17±1.36
ERN	14.17 ± 1.19	11.71~15.86	14.22 ± 1.45	9.87~21.14	0.85	13.25±1.51
KNPR	22.03 ± 3.15	15.89~30.25	21.17 ± 3.05	12.96~30.6	0.8	21.22±2.53
KNPE	317.09 ± 60.86	206.28~478.15	304.97 ± 54.95	153.11~529.13	0.8	n.a.
KWPE	74.13 ± 15.92	43.74~114.33	71.85 ± 10.86	40.93~100.99	0.74	n.a.
EW	92.9 ± 19.72	50.37~143.79	91.28 ± 15.81	41.26~171.62	0.77	n.a.
CW	18.65 ± 5.53	8.97~34.77	19.54 ± 4.16	9.74~38.77	0.81	15.83±4.06
LBT	1.12 ± 0.33	0.59~1.67	1.2 ± 0.43	0.32~2.77	0.73	n.a.

[a] The plot-mean broad-sense heritability.

[b] Phenotypes from an association mapping panel (AMP) consisting of 513 diverse maize inbred lines [67]. "n.a." means the phenotype was not available for this trait.

Table S2. Summary of genetic contributions to trait variance. The proportion of phenotypic variation explained by QTL identified via single marker GWAS (sQTL), haplotype GWAS (hGWAS) and the combined effect of both are listed for traits influencing flowering time, agronomic performance, or yield.

Class	Trait	sQTL	hQTL	all QTL
Flowering	DTT	0.38	0.59	0.66
Flowering	DTA	0.43	0.59	0.69
Flowering	DTS	0.29	0.41	0.54
Flowering	ATI	0.33	0.55	0.62
Flowering	SAI	0.18	0.49	0.53
Flowering	STI	0.37	0.66	0.71
Agronomic	EH	0.37	0.51	0.61
Agronomic	ELL	0.32	0.37	0.49
Agronomic	ELW	0.4	0.52	0.64
Agronomic	LNAE	0.39	0.54	0.65
Agronomic	LNBE	0.35	0.57	0.66
Agronomic	TBN	0.42	0.68	0.76
Agronomic	PH	0.33	0.48	0.59
Agronomic	TL	0.39	0.58	0.67
Yield	ED	0.09	0.48	0.5
Yield	EL	0.37	0.6	0.69
Yield	ERN	0.38	0.55	0.62
Yield	KNPR	0.2	0.58	0.62
Yield	KNPE	0.08	0.47	0.51
Yield	KWPE	0.08	0.45	0.47
Yield	EW	0.07	0.5	0.52
Yield	CW	0.19	0.45	0.5
Yield	LBT	0.03	0.32	0.33

Table S3. Known metabolites that are significantly correlated with ear leaf width. Correlation of metabolites measured in this study and ear leaf width, and significance of the correlation (p-value).

Metabolite	Correlation coefficient	p-value
Dehydroascorbic_acid	0.25	5.02E-02
DL_Pipecolic_acid	-0.34	2.81E-04
Fructose	-0.31	3.30E-02
Galactarate	-0.38	4.00E-03
Galactose	-0.24	4.54E-02
Maltose	0.23	5.09E-02
MeJA_Concentration	0.35	3.39E-03
Methyl_glucopyranoside	-0.23	3.07E-02
N-acetyl_D_glucosamine	-0.2	4.82E-02
Serine	-0.24	2.29E-02
Succinic_acid	-0.28	2.29E-02
Threitol	-0.37	1.18E-02
Trans_Zeatin	0.25	3.82E-02

Table S4. The information of *ZmGalOx1* type-I polymorphisms. Polymorphisms identified via re-sequencing of the CUBIC population.

Polymorphism	Position(bp)	Strand	Location	CDS_position	P	Annotation	Codon	Amino Acid
chr4.s_3470265	3470265	-1	EXON=3	42	3.29E-08	missense_variant	cAc/cCc	H/P
chr4.s_3467301	3467301	-1	INTRON=9	-	5.7E-09	splice_region_variant	-	-
chr4.s_3465291	3465291	-1	EXON=14	547	5.82E-12	missense_variant	caC/caG	H/Q
chr4.indel_3465235[a]	3465235	-1	EXON=14	566	8.8E-23	Frameshift, stop gained	cTg/cgt	L/R

[a] indicates the functional variant as the InDel_1/0.

Table S5. The allelic variations of 24 parents at the *ZmGalOx1* type-I polymorphisms. Polymorphisms are identified and summarized in Supp. Table 5.

Parent	chr4.s_3470265	chr4.s_3467301	chr4.s_3465291	chr4.indel_3465235[a]
HUANGC	TT	GG	CC	——
5237	GG	AA	GG	AA
81515	GG	AA	GG	AA
CHANG7-2	GG	AA	GG	AA
DAN340	TT	GG	GG	AA
E28	TT	GG	GG	AA
F349	TT	GG	CC	AA
H21	GG	AA	GG	AA
HYS	GG	AA	GG	AA
HZS	GG	AA	GG	AA
JI53	GG	AA	GG	AA
JI853	GG	AA	GG	AA
K12	TG	AA	GG	AA
LV28	GG	AA	GG	AA
LX9801	GG	AA	GG	AA
NX110	TG	AA	GG	AA
Q1261	TG	AA	GG	AA
SHUANG741	GG	AA	GG	AA
TY4	TG	AA	GG	AA
XI502	GG	AA	GG	AA
YUANFH	GG	AA	GG	AA
ZI330	TT	GG	GG	AA
ZONG3	TT	GG	GG	AA
ZONG31	TT	GG	CC	AA

[a] indicates the functional variant as the InDel_1/0.
